# Multiple Domain Associations within the Arabidopsis Immune Receptor RPP1 Regulate the Activation of Programmed Cell Death

**DOI:** 10.1371/journal.ppat.1005769

**Published:** 2016-07-18

**Authors:** Karl J. Schreiber, Adam Bentham, Simon J. Williams, Bostjan Kobe, Brian J. Staskawicz

**Affiliations:** 1 Department of Plant and Microbial Biology, University of California, Berkeley, Berkeley, California, United States of America; 2 School of Chemistry and Molecular Biosciences and Australian Infectious Diseases Research Centre, University of Queensland, Brisbane, Australia; 3 School of Biological Sciences, Faculty of Science and Engineering, Flinders University, Adelaide, Australia; 4 Division of Chemistry and Structural Biology, Institute for Molecular Bioscience, University of Queensland, Brisbane, Australia; The University of North Carolina at Chapel Hill, UNITED STATES

## Abstract

Upon recognition of pathogen virulence effectors, plant nucleotide-binding leucine-rich repeat (NLR) proteins induce defense responses including localized host cell death. In an effort to understand the molecular mechanisms leading to this response, we examined the *Arabidopsis thaliana* NLR protein RECOGNITION OF *PERONOSPORA PARASITICA*1 (RPP1), which recognizes the *Hyaloperonospora arabidopsidis* effector *ARABIDOPSIS THALIANA* RECOGNIZED1 (ATR1). Expression of the N-terminus of RPP1, including the Toll/interleukin-1 receptor (TIR) domain (“N-TIR”), elicited an effector-independent cell death response, and we used allelic variation in TIR domain sequences to define the key residues that contribute to this phenotype. Further biochemical characterization indicated that cell death induction was correlated with N-TIR domain self-association. In addition, we demonstrated that the nucleotide-binding (NB)-ARC1 region of RPP1 self-associates and plays a critical role in cell death activation, likely by facilitating TIR:TIR interactions. Structural homology modeling of the NB subdomain allowed us to identify a putative oligomerization interface that was shown to influence NB-ARC1 self-association. Significantly, full-length RPP1 exhibited effector-dependent oligomerization and, although mutations at the NB-ARC1 oligomerization interface eliminated cell death induction, RPP1 self-association was unaffected, suggesting that additional regions contribute to oligomerization. Indeed, the leucine-rich repeat domain of RPP1 also self-associates, indicating that multiple interaction interfaces exist within activated RPP1 oligomers. Finally, we observed numerous intramolecular interactions that likely function to negatively regulate RPP1, and present a model describing the transition to an active NLR protein.

## Author Summary

Many plant pathogens inject proteins known as effectors into the cells of their hosts in order to suppress host immune responses and promote pathogen growth. Over time, plants have evolved receptors, described as nucleotide-binding leucine-rich repeat (NLR) proteins, which recognize the activity of pathogen effectors and stimulate defense responses. Plant NLRs contain several domains that exhibit striking functional conservation with NLRs from other eukaryotes. Despite their important contribution to plant immunity, the molecular mechanisms that underlie effector recognition and subsequent immune activation by NLRs remain to be fully elucidated. Here, we focus on RPP1, an NLR from Arabidopsis that recognizes the oomycete effector ATR1. Using transient co-expression of proteins in plants, we demonstrate that recognition of ATR1 stimulates RPP1 oligomerization. This interaction involves multiple domains of RPP1 and is critical for immune activation. In the absence of ATR1, we documented interactions between domains within an individual RPP1 protein, likely occurring to prevent inappropriate immune activation. Finally, we examined differences between RPP1 alleles as well as structural data from animal NLRs to help identify specific amino acids that mediate interactions within and between RPP1 molecules. Collectively, these data allow us to propose a model for the activation of RPP1 following ATR1 recognition.

## Introduction

Through interactions with a plethora of phytopathogenic organisms, plants have evolved a sophisticated molecular surveillance system that involves nucleotide-binding leucine-rich repeat (NLR) immune receptors that recognize pathogen-derived virulence effector proteins. Effector recognition elicits a strong immune response that is associated with programmed cell death, known as the hypersensitive response (HR), which ultimately restricts pathogen proliferation [1,2]. Plant NLR proteins are generally modular in structure, with a C-terminal leucine-rich repeat (LRR) domain often being responsible for effector recognition, either directly or indirectly. A central nucleotide-binding domain with homology to Apaf-1, resistance proteins, and CED-4 (NB-ARC) is associated with nucleotide binding and hydrolysis, and acts as a molecular switch for NLR protein activation [3–6]. Upon activation, immune responses are thought to signal through the N-terminal region of the NLR protein, which may comprise an N-terminal coiled-coil (CC) or Toll/interleukin-1 receptor (TIR)-like domain. In the absence of pathogen-associated elicitors, NLR proteins are thought to be maintained in an inactive state by a number of intramolecular interactions [7,6]. Effector recognition by the LRR likely causes a conformational change that increases the accessibility of the NB-ARC domain to allow nucleotide exchange and subsequent NLR protein activation, although the specific structural changes that lead to this active state are unclear.

There is increasing evidence from the study of animal NLR immune receptors that intermolecular associations play a key role in the stimulation of immune responses. Similar to plant NLR proteins, these receptors include a nucleotide-binding domain flanked by a C-terminal ligand-sensing domain and an N-terminal signaling domain that recruits additional immunomodulatory proteins [8]. Following the detection of pathogen- or damage-derived signals, the assembly of higher-order complexes has been documented for proteins such as Apaf-1, NLRC4, CED-4, NALP1, and Dark [9–16]. Analyses of these inflammasome and apoptosome structures indicate that residues within and proximal to the nucleotide-binding domain are the primary regions of contact for oligomerization [13,14,17]. In plants, the only known example of ligand-dependent oligomerization involves the tobacco N protein, whose self-association is induced by recognition of the tobacco mosaic virus replicase [18]. Currently, it is not known whether this is a unique evolutionary development or representative of a common yet poorly characterized pathway for plant NLR protein activation.

In light of this uncertainty, we examined the *Arabidopsis thaliana* protein RPP1 (RECOGNITION OF *PERONOSPORA PARASITICA*1) [19], a TIR domain-containing NLR protein that recognizes the *Hyaloperonospora arabidopsidis* (formerly *Peronospora parasitica*) effector ATR1 (*ARABIDOPSIS THALIANA* RECOGNIZED1) [20]. This recognition is mediated by the LRR domain of RPP1, with both genetic and *in planta* biochemical evidence suggesting a direct mode of recognition [21,22]. Multiple alleles of RPP1 and ATR1 have been cloned and characterized, revealing significant allelic variation in the NLR protein/effector combinations that lead to cell death. For example, the Niederzenz (NdA) allele of RPP1 recognizes the Emoy2 allele of ATR1, while the Wassilewskija (WsB) allele of RPP1 recognizes the Emoy2, Maks9, and Emco5 ATR1 alleles. Neither RPP1 allele recognizes ATR1_Cala2. These polymorphic phenotypes have enabled an examination of the sequences that contribute to ATR1 recognition [22] and could also be used to dissect the activation of immune responses.

In this study, we sought to functionally characterize the domains of RPP1 with regards to cell death induction following effector recognition. Using transient expression of epitope-tagged proteins, we demonstrate that RPP1 oligomerizes in the presence of a recognized allele of ATR1. Co-immunoprecipitation and mutagenesis experiments were used to investigate the specific domains involved in pre-activation intramolecular interactions and post-activation intermolecular interactions. Phenotypic variation amongst RPP1 alleles and structural data from various animal immune receptors were used to identify specific amino acids that facilitate these protein-protein interactions. Based on these observations, we suggest a model of the molecular events underlying only the second documented example of effector-dependent NLR protein oligomerization in plants.

## Results

### Allelic variation in TIR domain autoactivity

**Fig 1:**
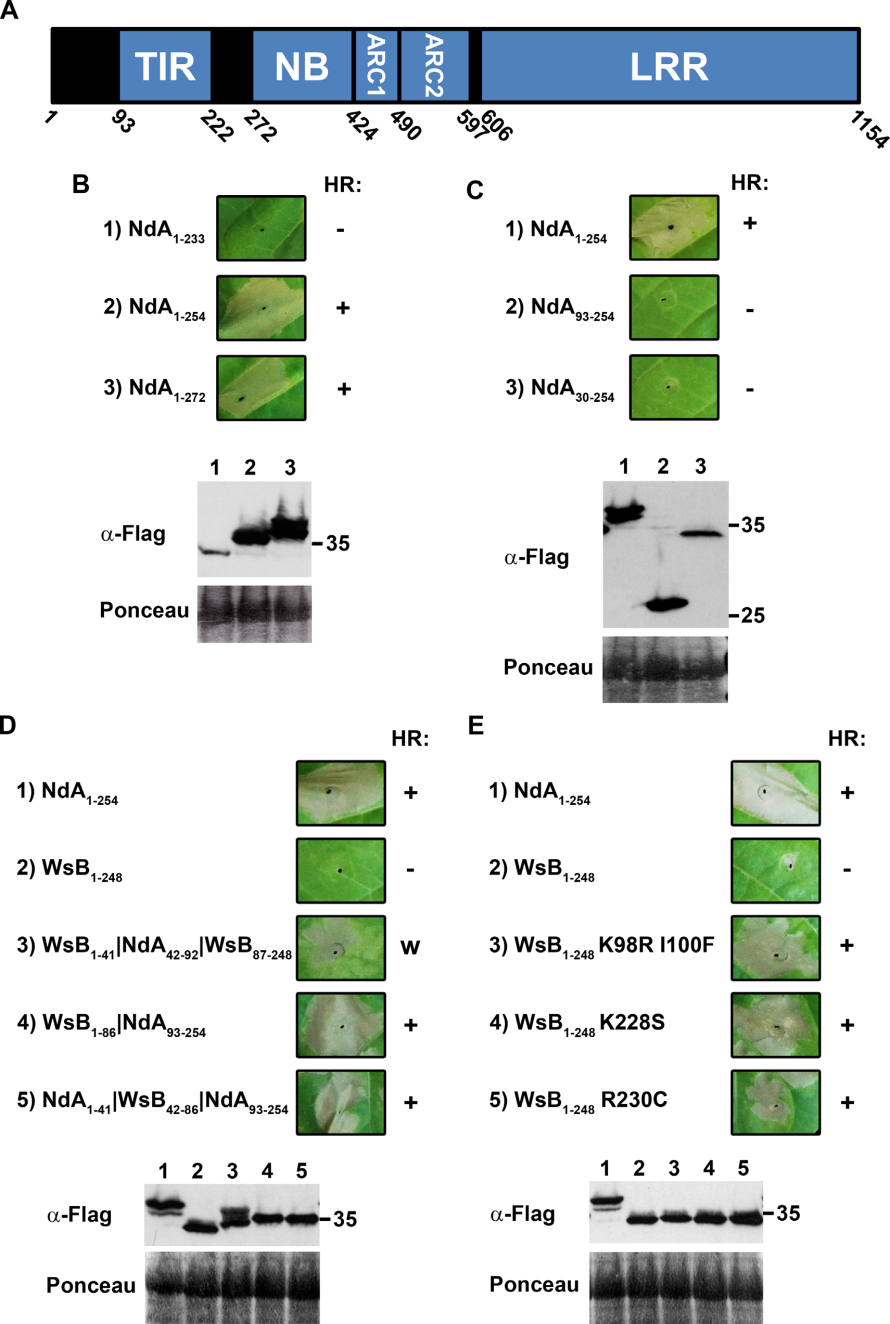
Identification of sequences required for RPP1 TIR domain autoactivity. (A) Schematic overview of the domain architecture of RPP1. Numbers indicate the amino acid position of predicted domain borders for the Niederzenz (NdA) allele of RPP1. (B,C) Determination of the minimal autoactive TIR domain from the NdA allele of RPP1. Both C-terminal (B) and N-terminal (C) truncations were examined for their ability to elicit an effector-independent hypersensitive response (HR). The specific amino acids comprising each construct are indicated in subscript. (D,E) HR phenotypes associated with chimeras or site-directed mutants of N-TIR domains from the NdA and Wassilewskija (WsB) alleles. Site-directed mutagenesis was guided by the amino acid alignment depicted in S2 Fig. Constructs were tested in *Nicotiana tabacum* via *Agrobacterium*-mediated transient expression and images were captured at 48 hours post-infiltration. HR phenotypes are scored as negative (-), weak (w), or strong (+). An α-Flag antibody was used to evaluate protein expression, while staining of RuBisCO with Ponceau S provided a loading control. The experiment was performed three times with similar results.

Our efforts to define the molecular events associated with cell death induction by RPP1 initially focused on the N-terminal region of this protein, which contains a TIR domain (Fig 1A). In other NLRs, the TIR domain executes the cell death response upon effector recognition [23,24]. When the first 254 amino acids of the NdA allele of RPP1 were transiently expressed in *Nicotiana tabacum*, an effector-independent HR developed within 24 hours (Fig 1B). This 254 aa region comprises the minimal autoactive sequence (Fig 1B and 1C) and includes the TIR domain preceded by an N-terminal region with no obvious homology to other functional domains, hereafter collectively referred to as “N-TIR”. Interestingly, although the WsB allele of RPP1 can recognize ATR1 and elicit cell death as a full-length protein, the equivalent N-TIR region from this allele did not exhibit autoactivity (Fig 1D). Quantification of electrolyte leakage induced by expression of these constructs confirmed the lack of autoactivity for the WsB allele (S1 Fig). To dissect this phenotypic polymorphism, we constructed chimeras of the two alleles and tested their ability to induce an HR. These analyses indicated that regions both within and N-terminal to the predicted TIR domain of RPP1_NdA conferred autoactivity (Fig 1D). In a WsB background, the inclusion of amino acids 42–92 from RPP1_NdA resulted in a relatively weak HR (Fig 1D). The autoactivity of the reciprocal chimera was not compromised (Fig 1D), indicating that the polymorphisms within this region were dispensable for cell death induction, although the N-terminal region itself was critical for autoactivity (Fig 1C).

To identify specific amino acids within the TIR domain that contribute to autoactivity, we performed site-directed mutagenesis based on amino acid polymorphisms between the NdA, WsB, and Estland (Est-1) alleles of RPP1 (S2 Fig). Est-1 was included because the sequence of its TIR domain closely resembles that of NdA [25], and it is also autoactive (S2 Fig). In a WsB background, the introduction of substitutions K98R I100F, K228S, or R230C (to mimic the NdA or Est-1 sequence) was sufficient to confer autoactivity (Fig 1E, S1 Fig). Constructs with K98R or I100F alone elicited a significantly weaker HR, indicating an additive effect of the two substitutions (S3 Fig). In an NdA background, substitution of the corresponding residues with alanines (R104A F106A) eliminated autoactivity, while replacement with the WsB residues (R104K F106I) had no effect on cell death (S4 Fig). Substitutions K234A and C236A also had no impact on the HR phenotype of the NdA N-TIR domain, although amino acid changes nearby (G229A Y230A) resulted in a loss of autoactivity (S4 Fig).

**Fig 2:**
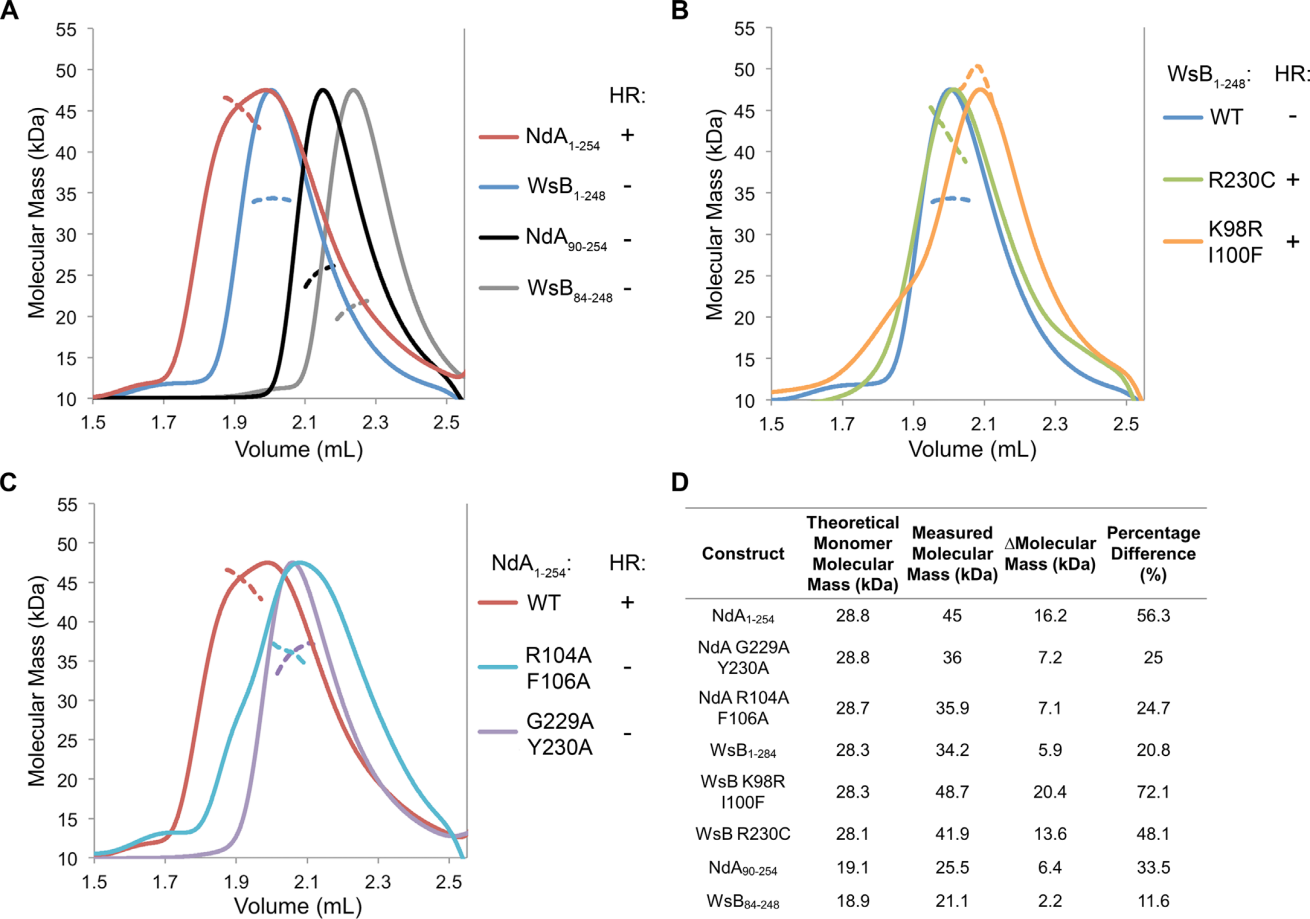
The autoactivity of the RPP1 N-TIR domain is correlated with self-association in solution. Purified (N-)TIR domain proteins from the Niederzenz (NdA) and Wassilewskija (WsB) alleles of RPP1 were analyzed by size-exclusion chromatography (SEC) coupled with multi-angle laser light scattering (MALS). For each sample, 175 μg of purified protein was separated on a Superdex Increase 200 5/150 GL SEC column and the molecular mass calculated across the elution peak. The colored solid line represents the normalized refractive index trace (arbitrary units) of the protein eluting from the SEC column. At the elution peak, the averaged molecular mass (kDa) of the proteins was calculated from the protein concentration (derived from the refractive index changes) and light scattering data. The averaged molecular masses across the elution peak are represented by dashed lines of the corresponding color. *In planta* hypersensitive response (HR) phenotypes are indicated for each construct by a “+” (autoactive) or “-”(non-autoactive). These phenotypes are documented in Fig 1, S2 and S4 Figs. (A) Comparison of the solution properties of RPP1 TIR domains with and without native RPP1 N termini. Numbers in the legend refer to the amino acids that comprise each protein sample. (B) Solution properties of the wild-type WsB N-TIR domain and the gain-of-autoactivity mutants, WsB R230C and WsB K98R I100F. (C) Solution properties of the wild-type NdA N-TIR domain and the loss-of-autoactivity mutants, NdA G229A Y230A and NdA R104A F106A. (D) Comparison of theoretical monomer molecular masses and the measured molecular masses for the proteins analyzed in (A-C).

Given that the autoactivity of some TIR domains is influenced by their ability to self-associate [23,26], we sought to determine if RPP1 exhibits the same behavior. Previous studies of NLR TIR domains, including that from RPP1, noted that self-association could not by detected by co-immunoprecipitation, likely due to weak and/or transient interactions [21,23,24]. As such, we employed a chromatography-based approach to evaluate TIR domain self-association. Size-exclusion chromatography (SEC) is commonly used to characterize protein size and oligomeric state indirectly by the comparison of retention times of proteins of interest with those of protein standards. However, the migration of proteins through SEC columns is influenced by a number of parameters including particle shape, flexibility and composition. For these reasons, we coupled SEC with multi-angle laser light scattering (MALS). The signal from MALS is directly related to the average molecular mass of proteins eluted from a SEC column, making it an ideal technique to investigate and compare the solution properties of purified recombinant TIR domain proteins. When we evaluated the TIR domain alone, the measured average molecular masses of NdA_90-254_ and WsB_84-248_ were 33.5% and 11.6% higher, respectively, than would be expected for a monomeric protein (Fig 2A and 2D). This suggests that both proteins self-associate in solution, although to a much greater degree for the NdA allele (Fig 2A). Interestingly, the inclusion of the N-terminal region preceding the TIR domain (NdA_1-254_ and WsB_1-248_) further increased the measured molecular mass relative to the expected molecular mass of a monomer. Again, this difference was much more striking for NdA compared to WsB (Fig 2D), suggesting that the N-TIR domain of NdA has a greater tendency to self-associate than that of WsB (Fig 2A). A change in retention time on the SEC column was not observed despite an increase in the measured molecular mass via MALS, which is indicative of a rapid, transient interaction of the protein in solution. In addition, gain-of-autoactivity N-TIR_WsB mutants (R230C and K98R I100F) exhibited significant increases in measured average molecular mass, similar to or even greater than the increase observed for wild-type NdA_1-254_ (Fig 2B and 2D). Conversely, large reductions in molecular mass were noted for loss-of-autoactivity N-TIR_NdA mutants (R104A F106A and G229A Y230A), such that they resembled the solution properties of wild-type WsB_1-248_ (Fig 2C and 2D). Altogether, these data indicate that the *in planta* autoactivity of the RPP1 N-TIR domain is correlated with its propensity for self-association in solution.

### The NB-ARC domain influences N-TIR domain autoactivity

**Fig 3:**
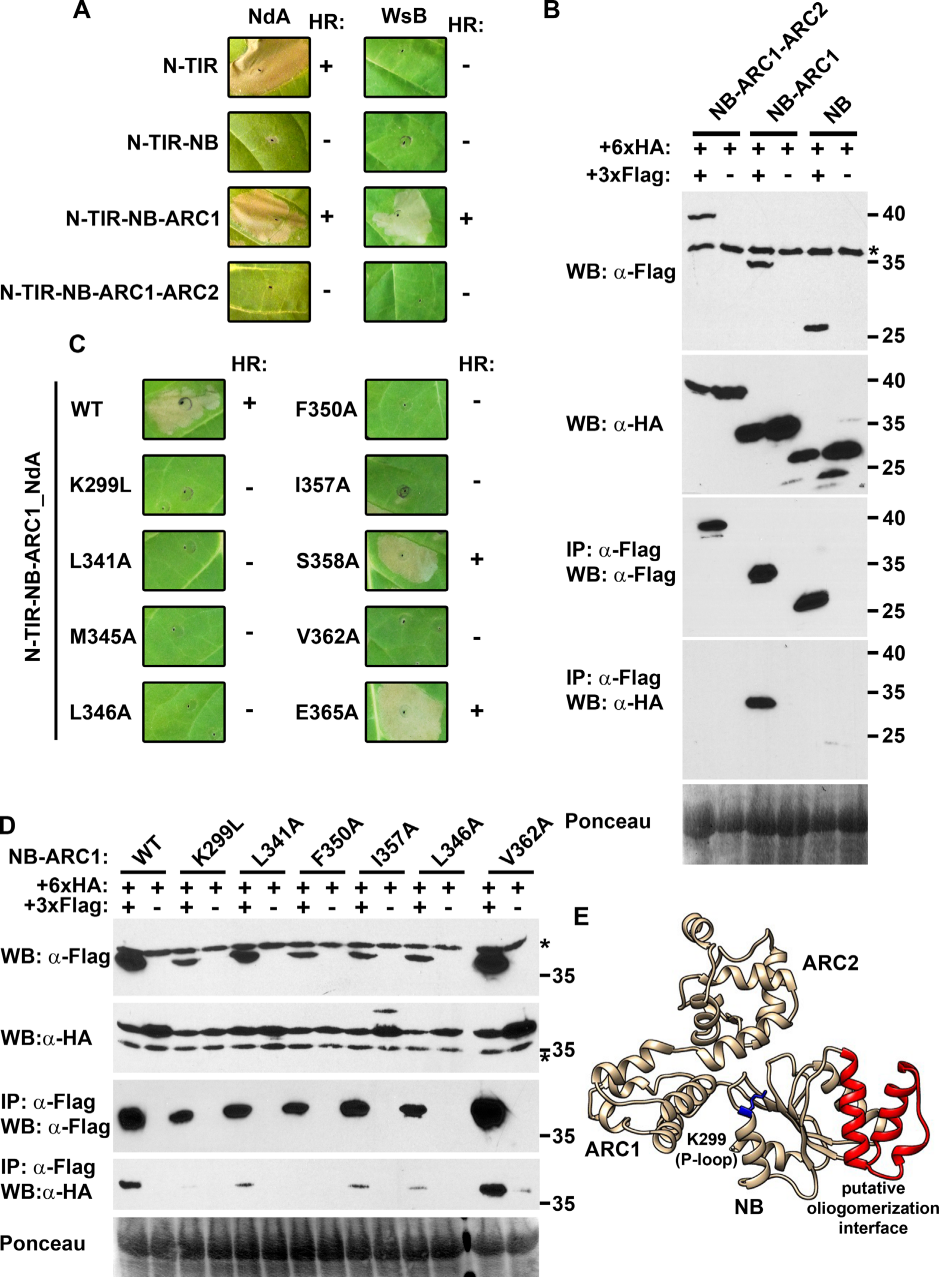
The NB-ARC domain influences N-TIR domain autoactivity. (A) Hypersensitive response (HR) phenotypes are altered by the successive addition of NB, ARC1, and ARC2 subdomains to the N-TIR domains of the RPP1 alleles Niederzenz (NdA) or Wassilewskija (WsB). Constructs were tested in *Nicotiana tabacum* via *Agrobacterium*-mediated transient expression and images were captured at 48 hours post-infiltration (hpi). The presence or absence of HR is indicated by a “+” or “-“, respectively. (B) Detection of self-association between NdA NB-ARC subdomain truncations by co-immunoprecipitation. Differentially epitope-tagged versions of NB, NB-ARC1, or NB-ARC1-ARC2 proteins were transiently expressed in *N*. *benthamiana* and samples were collected at 48 hpi for co-immunoprecipitation using α-Flag agarose beads. Asterisks indicate non-specific bands. Staining of RuBisCO with Ponceau S provides a loading control. (C) Site-directed mutagenesis of the NB subdomain compromises HR induction in an NdA N-TIR-NB-ARC1 background. Constructs were tested as in (A); protein expression for constructs from (A) and (C) is documented in S9 Fig. (D) Detection of self-association of NB-ARC1 mutants by co-immunoprecipitation, tested as in (B). Experiments were performed at least three times with similar results. (E) Predicted structure of the RPP1 NB-ARC domain derived from homology modeling using the Drosophila Dark protein (PDB:4v4l) as a template. The K299 residue within the P-loop is denoted in blue, while the putative oligomerization interface is highlighted in red. The specific residues at the putative oligomerization interface that were analyzed by mutagenesis are depicted in S10 Fig.

Despite the inability of the N-TIR domain from RPP1_WsB to elicit an HR, the full-length protein is capable of HR induction following recognition of the effector ATR1 [21,22]. This suggested that additional domains participate in cell death signaling. Indeed, when constructs comprising the N-TIR-NB-ARC1 region (aa 1–490 for NdA, aa 1–484 for WsB) were transiently expressed in *N*. *tabacum*, sequences from both the WsB and NdA alleles elicited an effector-independent HR (Fig 3A). Intriguingly, N-TIR-NB (NdA: aa 1–424; WsB: aa 1–418) and N-TIR-NB-ARC1-ARC2 (NdA: aa 1–597; WsB: aa 1–598) constructs were not autoactive for either allele. Measurements of electrolyte leakage corroborated these observations, although weak autoactivity was detected for the RPP1_NdA N-TIR-NB construct (S5 Fig). Further dissection of the ARC1 subdomain through C-terminal truncations revealed that a complete ARC1 was required for HR induction (S6 Fig).

Given the importance of N-TIR domain self-association for autoactivity, the contribution of the NB-ARC1 region may also depend upon homotypic interactions. Using co-immunoprecipitation, we demonstrated that the RPP1_NdA NB-ARC1 region (aa 255–490) could self-associate, while very weak interactions were observed for the NB region (aa 255–424) and no self-association was detected with NB-ARC1-ARC2 constructs (aa 255–597) (Fig 3B). Furthermore, self-association was observed with the autoactive N-TIR-NB-ARC1 proteins from alleles NdA and WsB (S7 Fig), providing further evidence that oligomerization significantly influences autoactivity. For the NB-ARC1 subdomain, co-immunoprecipitation yields from self-association experiments were consistently higher for the NdA allele relative to the WsB allele (S8 Fig), so subsequent analyses focused on RPP1_NdA.

The NB-ARC1 interactions that we observed are reminiscent of the *Caenorhabditis elegans* CED-4 protein, whose assembly into an apoptosome is mediated by intermolecular contacts on the surface of the α/β fold [13]. In RPP1, the homologous region is located in two α-helices of the NB subdomain (Fig 3E, highlighted in red, and S10 Fig). Alanine substitutions of surface-exposed residues on these helices generally eliminated the autoactivity of N-TIR-NB-ARC1 constructs, as did a K299L substitution at the predicted nucleotide-binding site, or P-loop (Fig 3C). One exception, however, was noted for the amino acid change E365A, which actually appeared to strengthen the HR (S11 Fig, S12 Fig). Co-immunoprecipitation experiments indicated that NB-ARC1 self-association was reduced in the loss-of-autoactivity mutants (Fig 3D), although *in planta* expression was also lower. This pattern was consistent aside from the N-TIR-NB-ARC1-inactivating substitution V362A, which retained self-association, accompanied by relatively high levels of protein expression. Overall, these data suggested that the NB subdomain, in cooperation with ARC1, is a key mediator of cell death induction.

### Effector-dependent oligomerization of full-length RPP1

**Fig 4:**
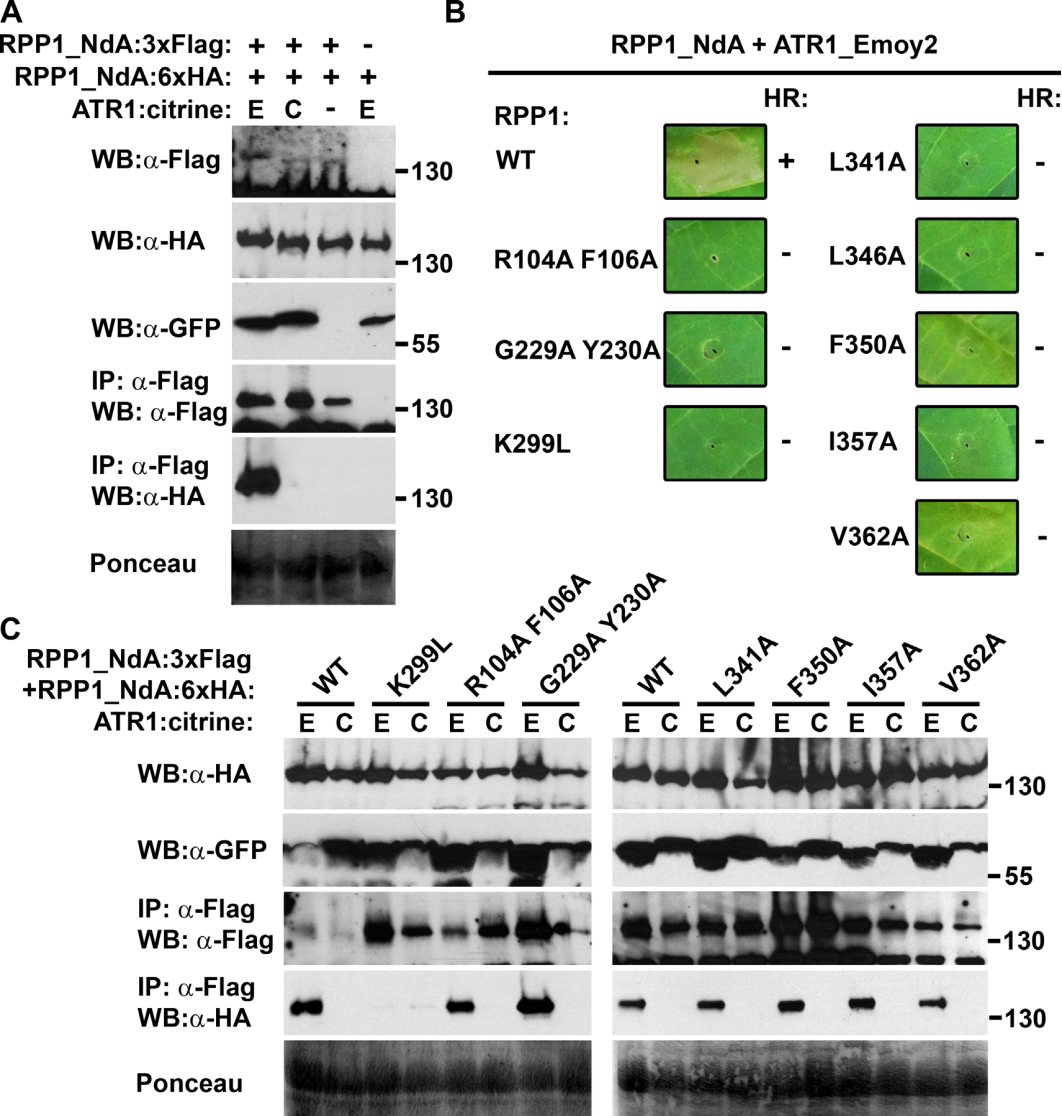
Effector-dependent oligomerization of full-length RPP1 is generally unaffected by mutations in the NB subdomain. (A) RPP1_NdA oligomerization is induced by co-expression with ATR1_Emoy2 (E) but not ATR1_Cala2 (C). Constructs were transiently expressed in *Nicotiana benthamiana* and samples were collected at 36 hours post-infiltration (hpi) for co-immunoprecipitation using α-Flag agarose beads. The expression of ATR1:citrine was detected with an α-GFP antibody. Staining of RuBisCO with Ponceau S provides a loading control. (B) Site-directed mutagenesis of the NB subdomain compromises effector-dependent HR induction by RPP1_NdA. Constructs were transiently co-expressed with ATR1_Emoy2 in *N*. *tabacum* and images were captured at 48 hpi. The presence or absence of HR is indicated by a “+” or “-“, respectively. Protein expression for these constructs is documented in S9 Fig. (C) Detection of self-association of RPP1_NdA NB subdomain mutants by co-immunoprecipitation, tested as in (A). Experiments were performed at least three times with similar results.

Following the characterization of effector-independent phenotypes in RPP1 domain truncations, we next sought to examine the function of these domains in the context of full-length RPP1. Transient co-expression of RPP1_NdA and ATR1 in *N*. *benthamiana* revealed RPP1 self-association in the presence of a recognized allele of ATR1 (ATR1_Emoy2) but not an unrecognized allele (ATR1_Cala2) (Fig 4A). The immunoprecipitated oligomer also included ATR1_Emoy2 (S13 Fig). Based on the oligomerization phenotype, we introduced the same substitutions into the putative oligomerization interface that were examined in the domain truncations, as well as substitutions within the TIR domain and P-loop. In most cases, these substitutions eliminated the effector-dependent HR (Fig 4B). Interestingly, effector-induced oligomerization was only significantly compromised in the P-loop (K299L) mutant (Fig 4C). Neither R104A F106A and G229A Y230A in the TIR domain, nor mutations on the putative oligomerization surface in the NB domain compromised self-association. Thus, mutations that affected dimerization of individual domains rarely compromised the oligomerization capacity of the full-length protein. Importantly, this indicates that RPP1 oligomerization is not an indirect byproduct of effector-induced cell death, but rather precedes the HR.

### Regulation of RPP1 intermolecular interactions

**Fig 5:**
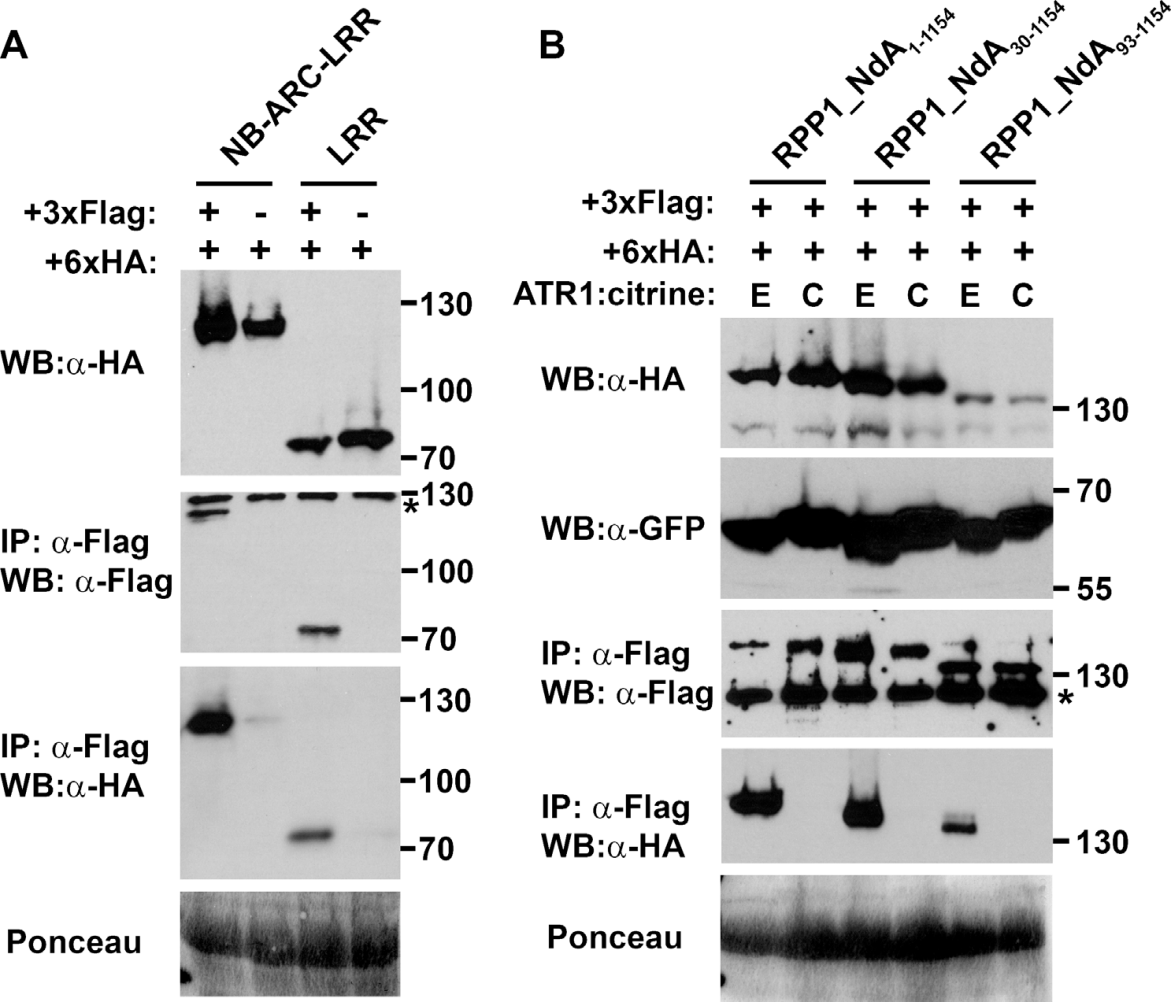
The TIR domain inhibits RPP1 self-association in the absence of the effector ATR1. (A) RPP1_NdA constructs lacking the TIR domain self-associate in an effector-independent manner. Differentially epitope-tagged versions of either NB-ARC-LRR or LRR proteins were transiently expressed in *N*. *benthamiana* and samples were collected at 48 hours post-infiltration (hpi) for co-immunoprecipitation using α-Flag agarose beads. Asterisks indicate non-specific bands. Staining of RuBisCO with Ponceau S provides a loading control. (B) The TIR domain alone is responsible for inhibiting RPP1 self-association, as N-terminal truncations up to the TIR domain retain effector-dependent self-association. Co-immunoprecipitation experiments were performed using differentially tagged proteins as in (A) except that tissue samples were collected at 36 hpi (E = ATR1_Emoy2, C = ATR1_Cala2). The expression of ATR1:citrine was detected with an α-GFP antibody. The specific amino acids comprising each construct are indicated in subscript. Experiments were performed three times with similar results.

Considering the differential impact of NB mutations on self-association between NB-ARC1 constructs versus full-length RPP1_NdA, it is possible that the LRR domain participates in the oligomerization event as well. Indeed, we detected LRR (aa 606–1154) and NB-ARC-LRR (aa 255–1154) self-association by co-immunoprecipitation, even in the absence of ATR1 (Fig 5A). The RPP1 LRR domain did not interact with the LRR domain of RPS2 (S14 Fig), suggesting that self-association is not due to non-specific protein-protein interactions. Notably, the addition of the TIR domain abolished this effector-independent self-association (Fig 5). Functionally, we observed that truncation of the first thirty amino acids of RPP1_NdA delayed the HR induced by ATR1_Emoy2 recognition, while removal of the first 92 amino acids (up to the predicted N-terminal border of the TIR domain) completely eliminated the cell death response (S15 Fig). Both of these truncations retained the capability for effector-dependent oligomerization (Fig 5B). As such, the TIR domain alone is an important negative regulator of RPP1 self-association, while additional N-terminal sequences are required for the function of the TIR domain as a positive regulator of cell death induction.

### Intramolecular interactions

**Fig 6:**
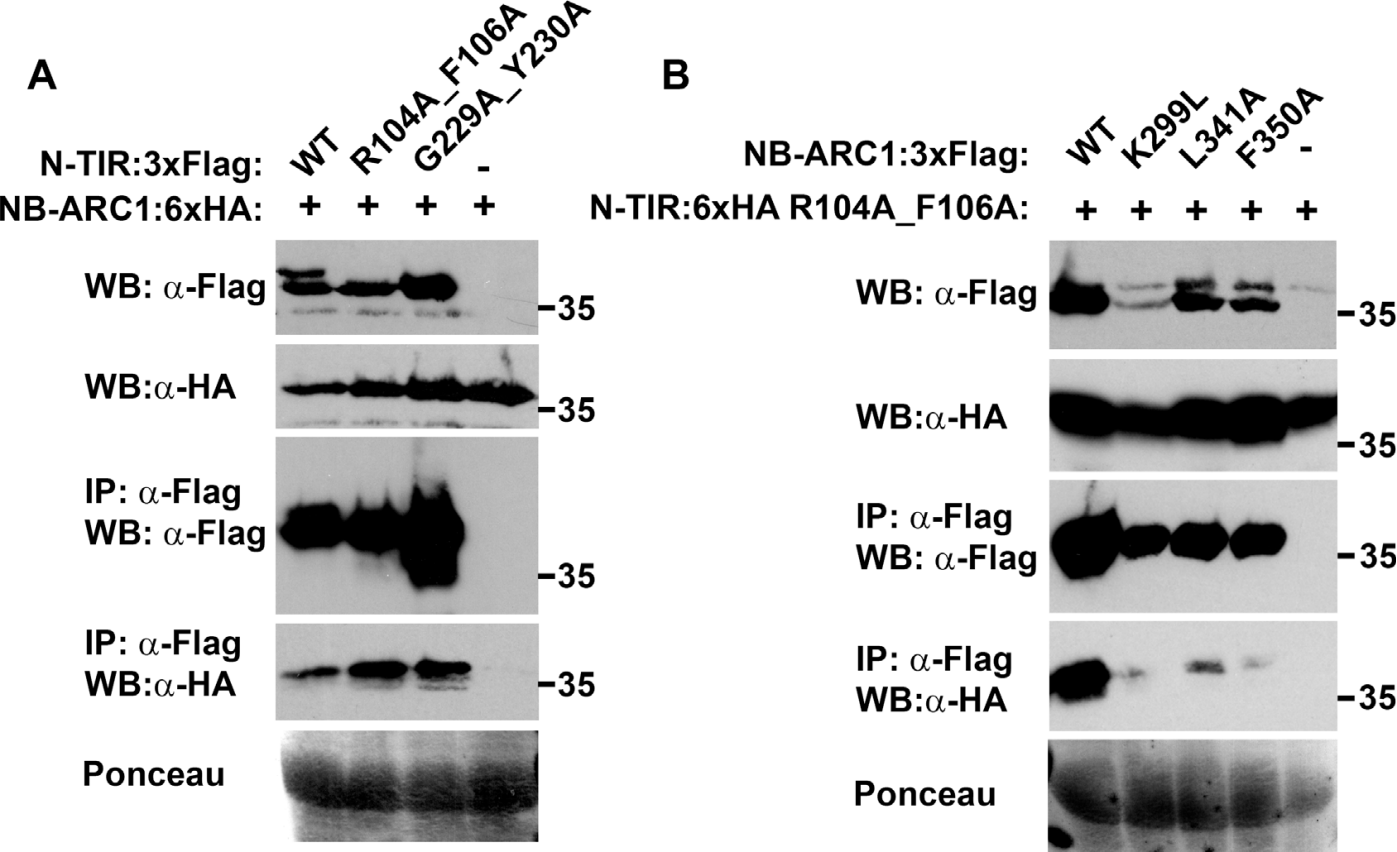
Evaluation of interactions between the N-TIR and NB-ARC1 domains of RPP1_NdA. (A) Non-autoactive N-TIR domain mutants associate with the NB-ARC1 subdomain. (B) Putative oligomerization interface mutants weaken the N-TIR:NB-ARC1 interaction. Constructs were transiently expressed in *Nicotiana benthamiana* and samples were collected at 36 hours post-infiltration (hpi) for (A) and 48 hpi for (B). Co-immunoprecipitations were performed using α-Flag agarose beads. Staining of RuBisCO with Ponceau S provides a loading control. Experiments were performed three times with similar results.

The demonstration of effector-induced oligomerization provided a snapshot of RPP1 in a post-activation state, but we also sought to clarify the domain organization of this protein prior to effector recognition. Through co-immunoprecipitation experiments we found that non-autoactive N-TIR domains, including the R104A F106A and G229A Y230A mutants of the NdA allele, interacted with the NB-ARC1 subdomain (Fig 6A). Less NB-ARC1 protein interacted with the wild-type N-TIR domain, although initial levels of N-TIR domain protein were somewhat lower as well. Using the NdA N-TIR R104A F106A mutant, we demonstrated that the NB subdomain comprised the minimal interaction region, although co-immunoprecipitation yields were slightly lower relative to NB-ARC1 (S16 Fig). Mutations in the P-loop (K299L) or at the putative oligomerization interface (L341A, F350A) appeared to reduce the binding of NB-ARC1 to TIR R104A F106A (Fig 6B), although input levels of NB-ARC1 mutant proteins were also lower.

**Fig 7:**
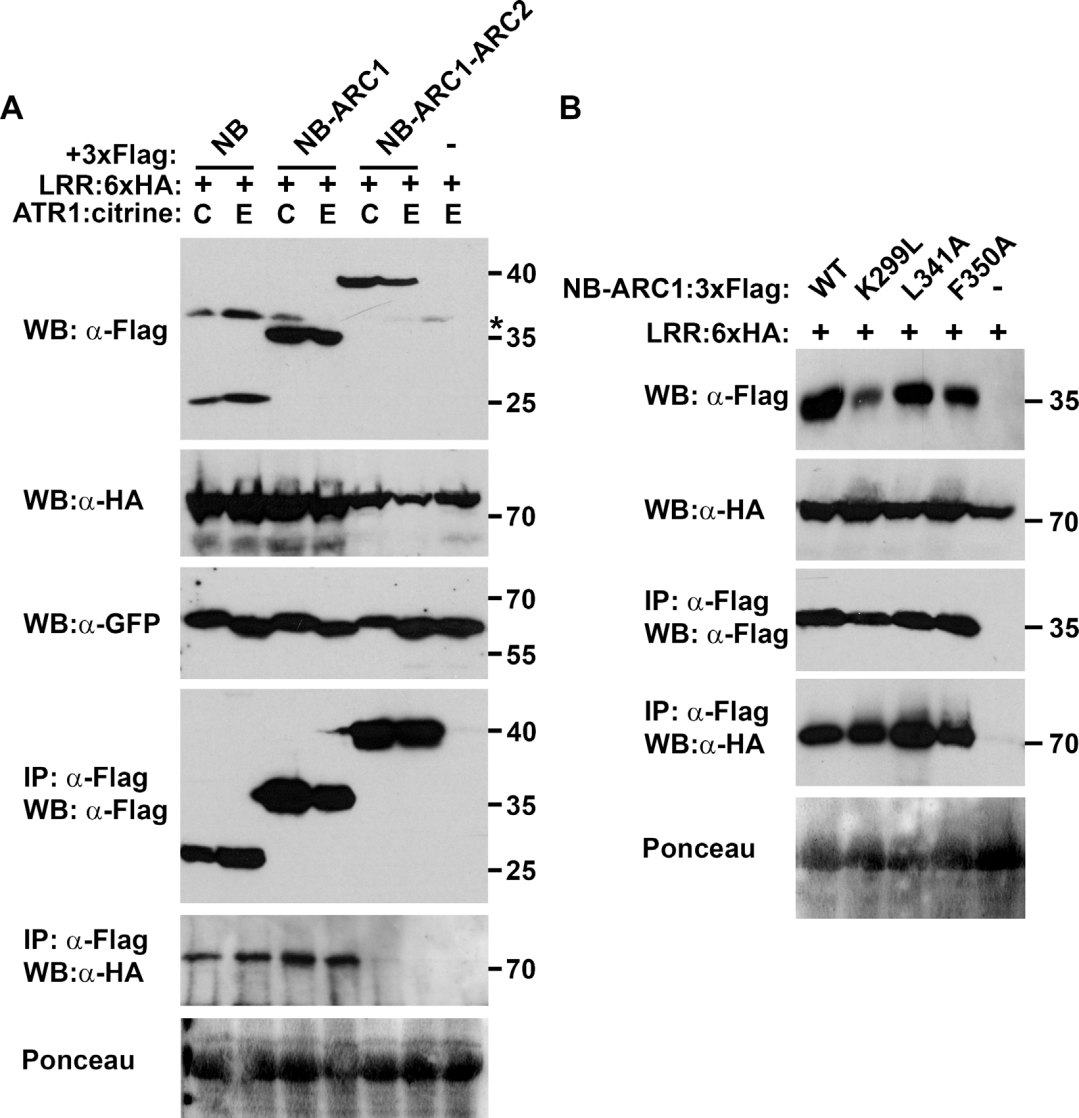
Evaluation of interactions between the LRR and NB(-ARC) domains of RPP1_NdA. The association between the NB(-ARC1) subdomain and the LRR is not disrupted by the presence of ATR1 (A) or by mutations at the putative oligomerization interface (B). Constructs were transiently expressed in *Nicotiana benthamiana* and samples were collected at 48 hours post-infiltration (E = ATR1_Emoy2, C = ATR1_Cala2). Co-immunoprecipitations were performed using α-Flag agarose beads. The expression of ATR1:citrine was detected with an α-GFP antibody. Asterisks indicate non-specific bands. Staining of RuBisCO with Ponceau S provides a loading control. Experiments were performed three times with similar results.

**Fig 8:**
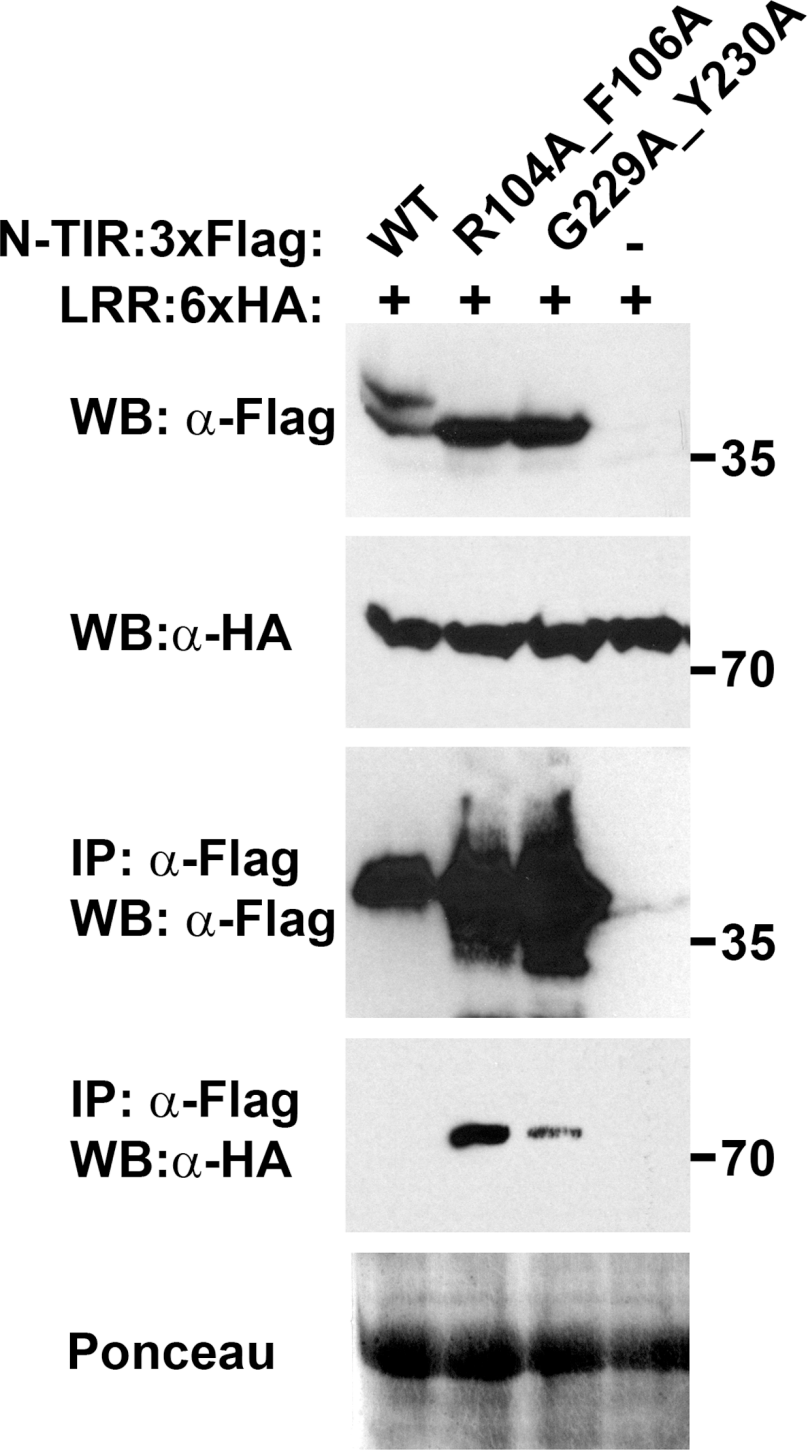
Non-autoactive N-TIR domain mutants associate with the LRR domain of RPP1_NdA. Constructs were transiently expressed in *Nicotiana benthamiana* and samples were collected at 36 hours post-infiltration. Co-immunoprecipitations were performed using α-Flag agarose beads. Staining of RuBisCO with Ponceau S provides a loading control. Experiments were performed three times with similar results.

Interactions were also detected between the NB-ARC1 subdomain and the LRR domain. The NB subdomain was again sufficient for this association, while little or no interaction was observed between the LRR and NB-ARC1-ARC2 (Fig 7A, S17 Fig). Given the proximity of ARC2 and the LRR within RPP1, this lack of binding was unexpected. We excluded the possibility that the location of the epitope tags precluded the interaction, because switching the tag to the N-terminus of NB-ARC1-ARC2 did not alter the co-immunoprecipitation results (S17 Fig). Focusing on the interaction between the LRR and NB-ARC1, we observed that the presence of ATR1 did not affect binding (Fig 7A), nor did mutations in the P-loop or at the putative oligomerization interface (Fig 7B). Finally, we detected interactions between the LRR domain and non-autoactive NdA N-TIR domain mutants, with reproducibly higher co-immunoprecipitation yields observed with the R104A F106A mutant versus G229A Y230A (Fig 8). Furthermore, the N-TIR R104A F106A protein associated with LRR and NB-ARC-LRR proteins in an effector-independent fashion (S18 Fig). Taken together, these data suggest that the N-TIR and LRR domains contact the NB domain at different interfaces, and that effector recognition may not induce a complete disruption of these interactions.

## Discussion

### Effector-independent cell death induction by the N-TIR domain of RPP1

Plant NLR proteins are characterized by a number of conserved domains whose collective activity yields tightly regulated immune responses. When expressed on its own, the N-TIR domain from RPP1_NdA elicited cell death even in the absence of ATR1. Similar autoactivity was noted for the TIR domains of RPS4 from Arabidopsis [24] and L6 from flax [23]. In both cases, the C-terminal border of the minimal autoactive region extended beyond the conserved TIR domain sequence predicted by Pfam [27] to include α-helix E. We also observed this with RPP1. Given the lower protein expression levels associated with further C-terminal truncations, the additional sequence likely affects protein stability.

Interestingly, N-TIR domains from different RPP1 alleles varied substantially in their autoactivity, allowing us to ascribe specific sequences to this phenotypic polymorphism. Using chimeric constructs, we noted that a region of 50 amino acids directly N-terminal to the predicted TIR domain from RPP1_NdA conferred weak autoactivity to an otherwise inactive background of RPP1_WsB. The functional role of this region is not known and its structure is predicted to be disordered, although our SEC-MALS analysis suggested that it contributes to TIR domain self-association (Fig 2). *In planta*, we noted that this N-terminal sequence is required for TIR domain autoactivity and for effector-dependent cell death induction by full-length RPP1_NdA. It is also worth noting that this region represents the most polymorphic sequence of the N-TIR domain between the NdA and WsB alleles, with 14 aa of insertions/deletions and 67% aa identity (versus 90% identity within the TIR domain).

The analysis of polymorphisms within the predicted TIR domain yielded a small number of residues with significant contributions to autoactivity. In a WsB background, the substitutions K98R I100F, K228S, or R230C enabled the elicitation of cell death at levels near that of the N-TIR domain from RPP1_NdA. Using the crystal structure of the L6 TIR domain [23] as a template for homology modeling, residue K98 is predicted to be exposed on the surface of the protein near α-helix A, while I100 may be located more in the interior of the protein (S2 Fig). Residues K228 and R230 are likely both surface-exposed between β-sheet E and x-helix E. The L6 crystal structure revealed a TIR domain dimerization interface spanning α-helix D to α-helix E, which Bernoux *et al*. [23] functionally interrogated by site-directed mutagenesis. The substitution R73A (near RPP1_WsB K98) abolished autoactivity, although the protein retained the ability to self-associate in a yeast two-hybrid assay. Conversely, an alanine substitution at D208 (corresponding to RPP1_WsB R230) resulted in weak autoactivity despite no detectable self-association. Additional TIR domain structures are available for RPS4 and RRS1, indicating homo- and heterodimerization interfaces at α-helices A and E [26]. Based on the SEC-MALS data, self-association of the RPP1 N-TIR domain may be influenced by both L6- and RPS4-like interfaces (Fig 2). More importantly, we demonstrated that allelic variation in N-TIR domain autoactivity is closely tied to the propensity for N-TIR domain self-association in solution. At least some degree of self-association was observed for all of the proteins that we analyzed, suggesting that some minimum level of association must be achieved for the activation of cell death. This may reflect a requirement for TIR domain dimers of sufficient stability to recruit adaptor proteins [28] for the execution of a cell death program.

### The regulatory role of the NB-ARC domain

The NB-ARC domain is generally considered to act as a molecular switch for NLR protein activation [3], but we have demonstrated an important role for this domain as a platform for oligomerization. This function is particularly important for RPP1_WsB, whose N-TIR domain exhibited autoactivity only with the inclusion of the NB and ARC1 subdomains. Krasileva *et al*. [21] previously showed that a C-terminal GFP fusion could facilitate autoactivity of the RPP1_WsB N-TIR domain, and that this effect was dependent upon the ability of GFP to dimerize. A similar dependence on GFP was noted for the CC domains from maize Rp1-D21 and Rp-1D [29]. A major difference, however, is that CC domain constructs containing even a small portion of the NB subdomain significantly weakened cell death induction, and the inclusion of the complete NB-ARC region blocked autoactivity. For the L6 TIR domain, the addition of NB or NB-ARC1 sequences reduced the cell death phenotype to a weak chlorosis which became even weaker, but not absent, with a TIR-NB-ARC1-ARC2 construct [23]. We observed more defined phenotypes with RPP1, in that an N-TIR-NB-ARC1 construct induced macroscopic cell death, while N-TIR-NB was extremely weak and an N-TIR-NB-ARC1-ARC2 construct was essentially inactive. As even a small truncation of the ARC1 subdomain eliminated autoactivity, it appears that the NB-ARC1 region comprises a discrete regulatory module. Insight into the potential function of this module was provided by co-immunoprecipitation experiments, which indicated that only NB-ARC1 proteins could self-associate. Again, this differentiates RPP1 from L6, where TIR domain self-association gradually diminished with the addition of NB and NB-ARC1 sequences, while TIR-NB-ARC1-ARC2 did not self-associate [23]. In either case, this provides further evidence for the negative regulatory function of the ARC2 subdomain as suggested previously by van Ooijen *et al*. [4].

Following the demonstration of self-association between NB-ARC1 proteins, we investigated the specific residues that influence this interaction. Substitution of a highly conserved lysine within the P-loop motif (K299L) eliminated NB-ARC1 self-association and N-TIR-NB-ARC1 autoactivity (Fig 3C). This residue was shown to be critical for ATP binding by the flax rust resistance protein M [5], and the loss of NLR protein function arising from mutations within the P-loop is well documented [30–34]. Structurally, the P-loop motif is buried within the NB subdomain, so its impact on protein self-association is likely exerted through conformational changes that are thought to accompany nucleotide exchange at this site [3].

The identification of residues at the physical protein interaction interface(s) of the NB-ARC1 region was guided by structural similarity with animal immune receptors. The activated *C*. *elegans* apoptosome comprises an octamer of CED-4 proteins forming a funnel-shaped structure, within which the α/β domains of each CED-4 member are closely aligned [13]. Structural data indicated that these interactions were stabilized by the stacking of hydrophobic side chains between neighboring α-helices. The importance of these residues for oligomerization was emphasized by mutagenesis within the homologous region of the mammalian protein Apaf-1, which eliminated apoptosome assembly. Likewise, we found that alanine substitutions of surface-exposed residues on the equivalent α-helices of RPP1_NdA reduced the self-association of NB-ARC1 proteins. While we currently lack the structural knowledge to confirm that this region acts as an interaction interface, it is worth noting that most of the surface-exposed residues are relatively hydrophobic, which is typical of many homodimerization interfaces [35–37].

An unusual observation was that the substitution E365A strengthened the intensity of cell death induced by an N-TIR-NB-ARC1 construct or by full-length RPP1_NdA co-inoculated with ATR1_Emoy2. While a glutamate-to-alanine substitution represents an increase in local surface hydrophobicity, similar increases in HR strength resulted from substitutions to the relatively hydrophilic amino acids glutamine and lysine (S11 Fig). As such, the effect may be more influenced by electrostatic forces, because all of these substitutions remove the negative charge at residue 365. In addition, the E365A substitution did not appear to increase the strength of self-association for NB-ARC1 proteins (S12 Fig), suggesting other mechanisms of cell death enhancement such as stabilization of the active state of RPP1. Regardless of the underlying mechanism, the ability to strengthen NLR-mediated immune responses in the absence of autoactivity could be useful for enhancing resistance in agricultural crops, as suggested by “sensitized” NB-ARC domain mutants of other plant NLRs [38–40].

### Effector-dependent oligomerization of full-length RPP1

While the assembly of higher-order immunoregulatory structures is well documented in animal systems [8,15], data from plant NLR proteins are relatively sparse and somewhat divergent. Effector-independent self-association was observed in members of the CC domain-containing family of NLR proteins, including RPS5, Rp1-D21, Rp1-D, Rp1-dp2, and MLA [29,41,42]. This behavior has not been observed in TIR domain-containing receptors, although RPS4 and RRS1 form a unique hetero-oligomer in the absence of recognized effectors [26]. Until now, the only example of elicitor-dependent oligomerization involved the tobacco N protein, whose homotypic interaction occurred only upon recognition of the helicase domain of the tobacco mosaic virus replicase [18]. Similar to our observations, mutations in the P-loop motif of N eliminated oligomerization, while mutations that abolished TIR domain autoactivity did not compromise self-association of full-length N proteins. Mestre and Baulcombe [18] also noted that mutations within the highly conserved “RNBS-A” motif disrupted the function of N while still permitting elicitor-dependent oligomerization. Our mutagenesis experiments in RPP1_NdA included residues L341 and L346, which are part of the RNBS-A motif and whose mutation yielded similar phenotypes. Overall, however, mechanistic interpretation of the results with the N protein was impaired by the inability to transiently express the NB-ARC domain *in planta*. We did not face such an obstacle with RPP1, and were able to demonstrate that residues within the RNBS-A motif and in the adjoining α-helix are involved in self-association of the NB-ARC1 subdomain. It is noteworthy that, while most of the substitutions retained some level of NB-ARC1 self-association, cell death induction was largely eliminated in both N-TIR-NB-ARC1 and full-length contexts. This may indicate that a certain threshold of association is required to activate cell death, and that substitutions of surface-exposed residues result in localized structural perturbations such that this threshold is not exceeded.

At the same time, the retention of effector-dependent oligomerization in mutants of full-length RPP1_NdA suggested that additional domains are involved in this interaction. Indeed, co-immunoprecipitation experiments revealed LRR self-association, even in the absence of ATR1. Similar homotypic interactions were shown for LRRs from RPS5 and Rp1-D21 [29,41], as might be expected given that the full-length proteins also exhibit effector-independent self-association. Full-length RPP1, however, likely exists in monomeric form prior to ATR1 recognition. By examining a series of N-terminal truncations, we found that the TIR domain is required to block effector-independent self-association of RPP1_NdA, likely by occluding the oligomerization interface on the NB domain.

### Intramolecular interactions

Resistance proteins control the induction of cell death programs and thus must be tightly regulated. For RPP1, we observed a series of conditional intramolecular interactions with potential negative regulatory functions. Firstly, interactions between the NB-ARC1 and N-TIR domains were most strongly detected with non-autoactive N-TIR domain mutants. Reduced protein accumulation of the wild-type NdA N-TIR domain likely influenced this result, although an appreciable quantity of the wild-type protein was immunoprecipitated. It is relevant to note that, while both CC:CC and CC:NB-ARC interactions were observed for RPS5 and Rp1-D21, the CC domain alone was not autoactive [29,41]. Based on our observations, it is possible that the affinity of N-TIR:NB-ARC1 binding is lower than that of either N-TIR domain self-association or adaptor binding, and that the outcome of these competing interactions is influenced by conformational changes that may accompany RPP1 activation. In this case, the reduced association between the NB-ARC and wild-type N-TIR domain proteins would reflect the active state of RPP1. This principle would also apply to the N-TIR:LRR interaction, which was similarly dependent on N-TIR domain-inactivating mutations. Furthermore, these data suggest that the interaction of the N-TIR domain with other RPP1 domains involves different interfaces than those required for N-TIR domain self-association. As a caveat, results obtained by transient co-expression of separate proteins may not reflect the interactions that occur in *cis*. Additional examples of this interaction from other TIR domain-containing NLR proteins would help to clarify these issues.

In addition to binding the N-TIR domain, we noted that the NB-ARC1 domain also interacts with the LRR domain of RPP1. Mutagenesis experiments revealed residues that differentially affected N-TIR:NB-ARC1 and LRR:NB-ARC1 interactions. Mutations in the NB P-loop motif significantly impaired N-TIR:NB-ARC1 binding but had little impact on the interaction of the LRR with NB-ARC1. A similar discrepancy was observed with the Rx protein from potato, where a P-loop mutation disrupted the CC:NB-ARC-LRR interaction but not the CC-NB-ARC:LRR interaction [43]. The P-loop motif is required for ATP binding [33,44] which is associated with NLR protein activation, perhaps through conformational changes [5,7]. If the CC/TIR and LRR domains interact with different NB-ARC surfaces, localized P-loop-dependent conformational changes in the NB-ARC domain could differentially affect these intramolecular interactions, especially if binding affinities also differ. Evidence for different intramolecular interaction surfaces comes from mutations at the putative oligomerization interface of the NB domain, which also reduced N-TIR:NB-ARC1 interactions to a much greater degree than LRR:NB-ARC1 interactions. Functionally, this supports a role for the TIR domain, and not the LRR, in preventing RPP1 self-association in the absence of ATR1.

For intramolecular interactions involving the LRR domain, we anticipated that effector binding might disrupt these associations, as seen in the NLR proteins Rx1 and Gpa2 [43,45]. We observed, however, that the presence of ATR1 did not affect either LRR:N-TIR or LRR:NB-ARC1 interactions. This may be a consequence of examining domain associations in *trans* rather than in *cis*, although it is difficult to bypass this limitation for *in planta* analyses. For the N-TIR domain, the use of a non-autoactive mutant may have allowed a more stable interaction with the LRR, as the wild-type N-TIR domain did not appear to interact with the LRR. It is also possible that the activation of RPP1 may involve a series of subtle conformational changes that preserve these intramolecular interactions, possibly through shifts in the specific residues bound by each domain.

**Fig 9:**
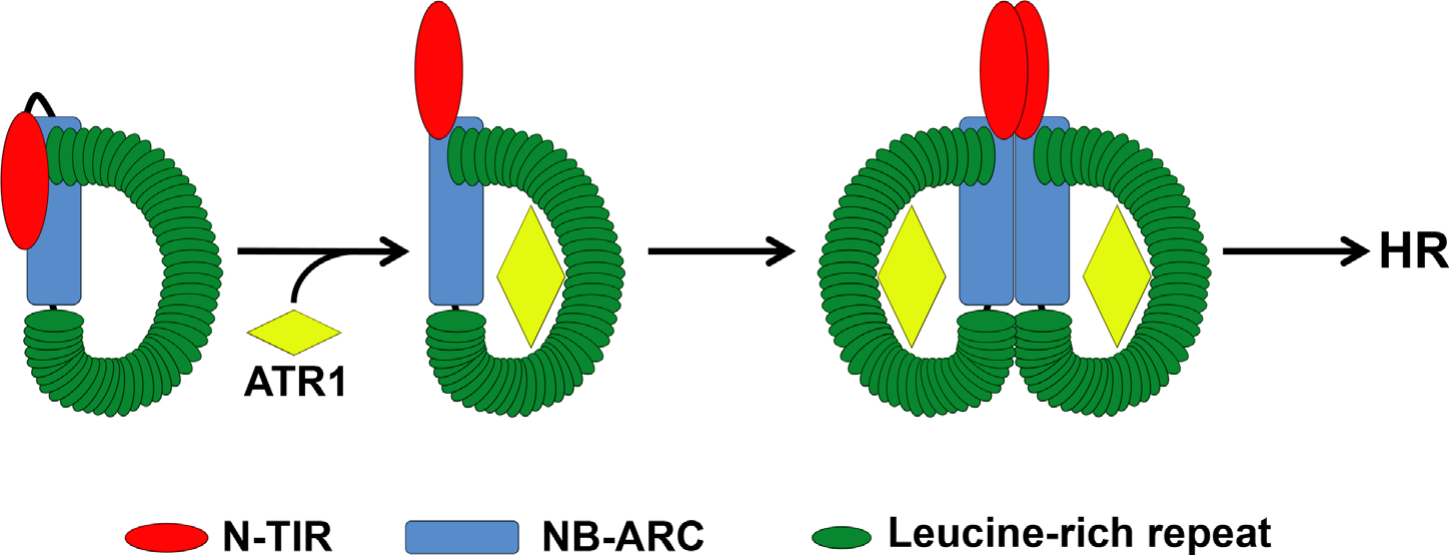
Proposed model of cell death activation by RPP1. In the absence of ATR1, RPP1 is likely maintained in a largely inactive state by a network of N-TIR:NB, NB:LRR, and N-TIR:LRR interactions. Note that the N-TIR:NB interaction occurs at the putative oligomerization interface to prevent effector-independent association. Binding of a recognized allele of ATR1 to the LRR domain may stabilize conformational transitions that reorient the N-TIR domain to expose the oligomerization interface. This allows RPP1 oligomerization via the NB domain, potentially stabilized by LRR:LRR interactions. The reorientation of the N-TIR domain also permits N-TIR domain self-association, which outcompetes N-TIR:NB interactions and ultimately triggers a cell death response (HR). While this model accounts for the effector-independent NB:LRR interaction, the specific interaction interface(s) could differ before and after RPP1 activation.

Overall, our data are compatible with a model (Fig 9) in which RPP1 is maintained in an inactive state by multiple intramolecular interactions. There may be some fluctuation between active and inactive states which is shifted towards the active state by ATR1 binding, as suggested by the recently proposed equilibrium-based switch activation model [6]. Stabilization of the active state by effector binding would be associated with some degree of conformational change sufficient to expose the N-TIR, NB, and LRR domains for self-association. These changes likely represent a subtle structural rearrangement that may only slightly alter the interfaces of intramolecular interactions, yet facilitate effector-dependent intermolecular associations. The specific details of this conformational transition remain unknown and await the elucidation of the structure of RPP1 as well as its interactions with other downstream signaling components.

## Materials and Methods

### Cloning

Constructs for transient expression were prepared using the primers listed in S1 Table. In general, sequences were first restriction-cloned into modified pENTR/D-TOPO (Thermo Fisher Scientific, Waltham, MA, USA) vectors so that inserts included 3’ sequences encoding either a 3xFlag or 6xhemagglutinin (HA) epitope tag. These entry clones were recombined into the pEarleygate vector pEG100 [46] using LR Clonase (Thermo Fisher Scientific). A construct for transient expression of the RPS2 LRR (aa 490–909, driven by a 35S promoter in vector pGWB14) was kindly provided by Douglas Dahlbeck (University of California, Berkeley). For protein expression in *Escherichia coli*, RPP1 TIR domain sequences were introduced into pLIC171 (T7 promoter, N-terminal His tag) by ligation-independent cloning [47]. All point mutations were introduced using a QuickChange Lightning Site-Directed Mutagenesis Kit (Agilent Technologies, Santa Clara, CA, USA) and the primers described in S1 Table. Mutagenesis experiments were guided in part by protein homology modeling using the Phyre2 web portal [48] combined with structure visualization using Chimera [49].

### Plant materials and growth conditions

*Nicotiana tabacum* var Turks and *Nicotiana benthamiana* were grown on potting soil in a controlled environment room with a 10-h photoperiod at 24°C. *Agrobacterium tumefaciens* GV3101 (pMP90) was grown at 28°C on Luria-Bertani agar media supplemented with 50 μg/mL gentamycin and 25 μg/mL kanamycin. For transient expression experiments, including co-immunoprecipitations, bacteria from plates were resuspended in 1 mL of induction media (10 mM MgCl_2_, 10 mM MES, and 150 μM acetosyringone, adjusted to pH 5.6 with KOH), diluted to OD_600_ = 0.45 with induction media, and incubated at room temperature for three hours. For all inoculations, leaves were pierced with a 22 gauge needle prior to infiltration with a 1 mL needleless syringe. Plants were subsequently maintained at room temperature under continuous light until tissue collection at 24 to 48 hours post-infiltration.

### Agrobacterium-mediated transient expression and co-immunoprecipitation

To detect transiently expressed proteins, two leaf discs were obtained from inoculated tissues using a #7 cork borer (1.2 cm diameter). The leaf discs were placed in a 1.7 mL SnapLock microfuge tube (Thermo Fisher Scientific) along with a 3 mm glass bead and frozen in liquid nitrogen. Samples were homogenized for 1.5 min in a chilled aluminum block with a Mini-Beadbeater-96 (BioSpec Products, Inc., Bartlesville, OK, USA) and suspended in 130 μL of modified Laemmli buffer (0.24 M Tris-Cl, pH 6.8, 6% SDS, 30% glycerol, 16% β-mercaptoethanol, 0.006% bromophenol blue, and 10 M urea). Samples were vortexed for 30 s, boiled for 5 min, centrifuged at maximum speed for 1 min in a microcentrifuge at room temperature, and the supernatants were transferred to 1.5 mL microfuge tubes. Generally, 12 μL of each sample was separated on 7% or 12.5% discontinuous SDS-PAGE gels and transferred to a nitrocellulose membrane (GVS North America, Sanford, ME, USA). Antibodies for protein detection included monoclonal ANTI-FLAG M2-Peroxidase (Sigma-Aldrich, St. Louis, MO, USA), anti-HA-Peroxidase (clone 3F10; Sigma-Aldrich), mouse monoclonal anti-GFP (EMD Millipore, Billerica, MA, USA), and monoclonal ANTI-FLAG M2 antibody (Sigma-Aldrich). The latter two antibodies were used in conjunction with a goat-anti-mouse HRP secondary antibody. Antibody binding was visualized with either SuperSignal West Pico or SuperSignal West Femto Maximum Sensitivity Chemiluminescent Substrate (Thermo Fisher Scientific).

Co-immunoprecipitation experiments were conducted essentially as described by Krasileva *et al*. [21] with some modifications. One gram (fresh weight) of leaf tissue was frozen in liquid nitrogen and homogenized with a mortar and pestle, then transferred to a prechilled mortar containing 2 mL of protein extraction buffer (50 mM Tris-HCl, pH 7.5, 150 mM NaCl, 0.1% Triton X-100, 0.2% Nonidet P-40, 6 mM β-mercaptoethanol, and cOmplete ULTRA Protease Inhibitor Cocktail [Sigma-Aldrich]) per gram of tissue and homogenized with a prechilled pestle for an additional minute. The homogenate was transferred to 1.5 mL microfuge tubes and centrifuged at 16,000 x g for 20 min at 4°C. Subsequently, 1.4 mL of the supernatant (approximately 70% of the total extract) was transferred to a 1.5 mL microfuge tube containing 15 μL of ANTI-FLAG M2 Affinity Gel (Sigma-Aldrich) and incubated for three hours on a rotator at 4°C. The tubes were centrifuged at 1,500 x g for 10 sec at 4°C, resuspended in 0.5 mL of extraction buffer, and centrifuged in the same manner again, repeating the process for a total of three washes. The beads were resuspended in 50 μL of Laemmli buffer, boiled for 5 min, then centrifuged for 1 min at maximum speed in a microfuge at room temperature. Twelve microliters of each sample was separated on 7% or 12.5% discontinuous SDS-PAGE gels and transferred to a nitrocellulose membrane. Immunodetection of co-immunoprecipitation samples was conducted as described above. For figures in which Flag-tagged inputs are not shown, levels of these proteins were below the threshold of detection with the α-Flag antibody.

### Measurement of electrolyte leakage

To quantify the extent of cell death elicited by various constructs, *Agrobacterium* suspensions were prepared as described above and infiltrated into *N*. *tabacum* leaves such that each technical replicate comprised all constructs infiltrated into sections of the same leaf. When macroscopic HR symptoms became visible (usually within 28 hours post-infiltration), four leaf discs were collected using a #7 cork borer (total area ≈ 4.5 cm^2^) and floated on 5 mL of distilled water for 30 min. Leaf discs were then transferred to polypropylene culture tubes containing 6 mL of distilled water and placed under continuous light at room temperature. Twenty-four hours later, electrolyte leakage was measured using a Thermo Orion Model 105A Plus Conductivity Meter (Thermo Fisher Scientific).

### Analysis of protein self-association by size-exclusion chromatography and multi-angle laser light scattering

The expression of RPP1 (N-)TIR domain proteins in *E*. *coli* BL21 (DE3) cells was induced overnight at 20°C via autoinduction [50]. Proteins were purified by immobilised metal-affinity chromatography with the use of a 6x histidine tag. The tag was cleaved from the proteins with TEV protease overnight at 4°C before separation by size-exclusion chromatography (SEC) on a Superdex S75 26/60 HiLoad column (GE Healthcare, Silverwater, NSW, Australia) in a buffer containing 10 mM HEPES pH 7.5, 150 mM NaCl, and 1 mM dithiothreitol (DTT).

For protein molecular mass determination, 175 μg of purified protein was separated on a Superdex Increase 200 5/150 GL SEC column (GE Healthcare) in a buffer containing 10 mM HEPES pH 8.0, 150 mM NaCl, 1 mM DTT, with laser light scattering measured by a Dawn Heleos II 18-angle light-scattering detector coupled with an Optilab TrEX refractive index detector (Wyatt Technology, Santa Barbara, CA, USA). Astra6.1 software (Wyatt Technology) was used to perform molecular mass calculations. Molecular mass is reported at the protein elution peak (corresponding to the refractive index peak) from the SEC column, where the signal from light-scattering is strong. Estimations of molecular mass were performed across the protein elution peak, with refractive increment (dn/dc values) fixed at 0.186 mL/g, with the assumption that dn/dc is invariable for unmodified proteins [51].

### Sequence accession numbers

Sequence data for the genes examined in this study can be retrieved from the National Center for Biotechnology Information using the following accession numbers: RPP1_NdA (HM209027), RPP1_WsB (AAC72978), ATR1_Emoy2 (AAX51198), ATR1_Cala2 (AAX51204), and RPS2 (AAA21874).

## Supporting Information

S1 Fig Quantification of electrolyte leakage induced by allelic chimeras and site-directed mutants of the RPP1 N-TIR domain. Data in (A) and (B) correspond to the constructs tested in (D) and (E) of Fig 1, respectively. The specific amino acids comprising each construct are indicated in subscript. Leaf discs representing approximately 4.5 cm^2^ of tissue were collected at approximately 28 hours post-infiltration and electrolyte concentration (conductivity) was measured 24 hours after collection. Error bars indicate standard deviation, and letters above data points indicate statistical significance groups as determined by pairwise Student’s t-tests (α = 0.05). Experiments were performed at least three times with similar results.
(TIF)

S2 Fig Analyses of polymorphic sequences within the RPP1 N-TIR domain. (A) Amino acid alignment of N-TIR domain sequences from the Niederzenz (NdA), Wassilewskija (WsB), and Estland-1 (Est-1) alleles of RPP1. The TIR domain borders predicted by Pfam are delimited by square brackets, and polymorphic amino acids with potential relevance to protein function are highlighted in red. Residues shown to influence autoactivity are indicated with arrowheads. (B) Functionally relevant polymorphic residues are also indicated on a putative structure of the RPP1_WsB TIR domain, derived by homology modeling using structural data from the L6 TIR domain (PDB: 3OZI). Relevant α-helices are also annotated. (C) Autoactivity of N-TIR domains from different RPP1 alleles. Constructs were tested in *Nicotiana tabacum* via *Agrobacterium*-mediated transient expression and images of hypersensitive response (HR) phenotypes were captured at 48 hours post-infiltration. The presence or absence of HR is indicated by a “+” or “-“, respectively. The specific amino acids comprising each construct are indicated in subscript. (D) An α-Flag antibody was used to evaluate protein expression, while staining of RuBisCO with Ponceau S provided a loading control. The experiment was performed three times with similar results.
(TIF)

S3 Fig Additivity of K98R and I100F substitutions in conferring autoactivity to the N-TIR domain from the WsB allele. Constructs were tested in *Nicotiana tabacum* via *Agrobacterium*-mediated transient expression and images of hypersensitive response (HR) phenotypes were captured at 48 hours post-infiltration. HR phenotypes are scored as negative (-), weak (w), or strong (+). The specific amino acids comprising each construct are indicated in subscript. An α-Flag antibody was used to evaluate protein expression, while staining of RuBisCO with Ponceau S provided a loading control. The experiment was performed three times with similar results.
(TIF)

S4 Fig Identification of loss-of-function mutations in the N-TIR domain from the NdA allele. Constructs were tested in *Nicotiana tabacum* via *Agrobacterium*-mediated transient expression and images of hypersensitive response (HR) phenotypes were captured at 48 hours post-infiltration. The presence or absence of HR is indicated by a “+” or “-“, respectively. The specific amino acids comprising each construct are indicated in subscript. An α-Flag antibody was used to evaluate protein expression, while staining of RuBisCO with Ponceau S provided a loading control. The experiment was performed three times with similar results.
(TIF)

S5 Fig The NB-ARC domain influences N-TIR domain autoactivity in RPP1_NdA. Leaf discs representing approximately 4.5 cm^2^ of tissue were collected at approximately 28 hours post-infiltration and electrolyte concentration (conductivity) was measured 24 hours after collection. Error bars indicate standard deviation, and letters above data points indicate statistical significance groups as determined by pairwise Student’s t-tests (α = 0.05). The experiment was performed three times with similar results.
(TIF)

S6 Fig The ARC1 domain comprises a discrete functional module in the regulation of N-TIR domain autoactivity. (A) Hypersensitive response (HR) phenotypes associated with C-terminal truncations of an N-TIR-NB-ARC1 construct from the NdA allele. The amino acids at the C-terminus of each truncation are indicated in subscript. Constructs were tested in *Nicotiana tabacum* via *Agrobacterium*-mediated transient expression and images were captured at 48 hours post-infiltration. HR phenotypes are scored as negative (-), very weak (vw), weak (w), or strong (+). (B) An α-Flag antibody was used to evaluate protein expression, while staining of RuBisCO with Ponceau S provided a loading control. Experiments were performed at least three times with similar results. (C) Predicted structure of the RPP1 NB-ARC domain based on homology modeling using the Drosophila Dark protein (PDB:4v4l) as a template. The three subdomains of this region are highlighted and the locations of each truncation tested in (A) are indicated by arrows.
(TIF)

S7 Fig Autoactive RPP1 N-TIR-NB-ARC1 proteins are capable of self-association. Differentially epitope-tagged N-TIR-NB-ARC1 proteins from the Niederzenz (N) and Wassilewskija (W) alleles of RPP1 were transiently expressed in *Nicotiana benthamiana* and samples were collected at 48 hours post-infiltration for co-immunoprecipitation using α-Flag agarose beads. Staining of RuBisCO with Ponceau S provides a loading control. The experiment was performed three times with similar results.
(TIF)

S8 Fig Co-immunoprecipitation yields from self-association experiments using the NB-ARC1 subdomain of RPP1 from alleles Niederzenz (N) and Wassilewskija (W). Constructs were transiently expressed in *Nicotiana benthamiana* and samples were collected at 48 hours post-infiltration for co-immunoprecipitation using α-Flag agarose beads. Staining of RuBisCO with Ponceau S provides a loading control. The experiment was performed three times with similar results.
(TIF)

S9 Fig *In planta* expression of constructs used for hypersensitive response assays. (A) N-TIR domain addition constructs tested in Fig 3A, including sequences from the RPP1 alleles Niederzenz (NdA) and Wassilewskija (WsB). (B) N-TIR-NB-ARC1 (NdA) constructs tested in Fig 3C. (C) Full-length RPP1 (NdA) constructs tested in Fig 4. Constructs were transiently expressed in *Nicotiana benthamiana* and samples were collected at 24 hours post-infiltration (hpi) for (A) and (B) or 48 hpi for (C). Staining of RuBisCO with Ponceau S provides a loading control. The experiments were performed three times with similar results.
(TIF)

S10 Fig Location of residues of interest at the putative oligomerization interface of the RPP1 NB subdomain. The α-helices that comprise the oligomerization interface of the *Caenorhabditis elegans* CED-4 protein (PDB: 2A5Y) are shown on the left, with residues that contribute to oligomerization highlighted in red. The corresponding region of RPP1 is shown at center; residues required for induction of the hypersensitive response are highlighted in red, while those that were dispensable for this response are depicted in green. The predicted structure of the RPP1 NB-ARC domain is shown on the right and is derived from homology modeling using the Drosophila Dark protein (PDB:4v4l) as a template. The putative oligomerization interface region is highlighted in red. Note that the enlarged image of this region is rotated approximately 90° counterclockwise on the y-axis for improved visibility of the residues of interest.
(TIF)

S11 Fig Amino acid substitutions at glutamate 365 (E365) in RPP1_NdA enhance cell death phenotypes. (A) Hypersensitive response phenotypes elicited by N-TIR-NB-ARC1 constructs or by co-expression of full-length RPP1_NdA and ATR1_Emoy2. Constructs were tested in *Nicotiana tabacum* via *Agrobacterium*-mediated transient expression, using a range of inoculum concentrations (as measured by OD_600_) to allow a comparison of the strength of cell death induction by wild-type (WT) and E365 substitution mutants. Images were captured at 48 hours post-infiltration (hpi). (B) An α-Flag antibody was used to evaluate protein expression, while staining of RuBisCO with Ponceau S provided a loading control. (C) Quantification of electrolyte leakage induced by various N-TIR-NB-ARC1 constructs. Leaf discs representing approximately 4.5 cm^2^ of tissue were collected at approximately 28 hpi and electrolyte concentration (conductivity) was measured 24 hours after collection. The non-autoactive F350A mutant and autoactive N-TIR domain were included as negative and positive controls, respectively. Error bars indicate standard deviation, and letters above data points indicate statistical significance groups as determined by pairwise Student’s t-tests (α = 0.05). Experiments were performed three times with similar results.
(TIF)

S12 Fig Mutagenesis of glutamate 365 (E365) does not increase NB-ARC1 self-association. Constructs were transiently expressed in *Nicotiana benthamiana* and samples were collected at 48 hours post-infiltration for co-immunoprecipitation using α-Flag agarose beads. Asterisks indicate non-specific bands. Staining of RuBisCO with Ponceau S provides a loading control. The experiment was performed three times with similar results.
(TIF)

S13 Fig ATR1_Emoy2 is present in the effector-dependent RPP1_NdA oligomer. Differentially epitope-tagged RPP1_NdA proteins were transiently co-expressed with either ATR1_Emoy2 (E) or ATR1_Cala2 (C) in *Nicotiana benthamiana* and samples were collected at 36 hours post-infiltration for co-immunoprecipitation using α-Flag agarose beads. Due to a non-specific signal at the expected molecular weight of ATR1:citrine when the elution fraction was probed with an α-GFP antibody, HA:ATR1:citrine constructs were used and ATR1 expression detected with an α-HA antibody. Staining of RuBisCO with Ponceau S provides a loading control. The experiment was performed three times with similar results.
(TIF)

S14 Fig The LRR domain of RPP1_NdA does not interact non-specifically with an unrelated LRR. While self-association of the RPP1_NdA LRR is detected, this protein does not interact with the LRR domain of RPS2. Constructs were transiently expressed in *Nicotiana benthamiana* and samples were collected at 48 hours post-infiltration for co-immunoprecipitation using α-Flag agarose beads. Staining of RuBisCO with Ponceau S provides a loading control. Experiments were performed three times with similar results.
(TIF)

S15 Fig The first thirty amino acids of RPP1_NdA are not required for cell death induction. Constructs were tested in *Nicotiana tabacum* via *Agrobacterium*-mediated transient co-expression with ATR1_Emoy2, and images of hypersensitive response phenotypes were captured at 48 and 72 hours post-infiltration (hpi). The constructs included RPP1_NdA_1-1154_ (1), RPP1_NdA_30-1154_ (2), and RPP1_NdA_93-1154_ (3), where the specific amino acids comprising each construct are indicated in subscript. An α-HA antibody was used to evaluate protein expression, and the expression of ATR1_Emoy2:citrine was detected with an α-GFP antibody. Staining of RuBisCO with Ponceau S provided a loading control. The experiment was performed three times with similar results.
(TIF)

S16 Fig The NB subdomain is sufficient for interaction with the N-TIR domain of RPP1_NdA. Constructs were transiently expressed in *Nicotiana benthamiana* and samples were collected at 48 hours post-infiltration for co-immunoprecipitation using α-Flag agarose beads. Staining of RuBisCO with Ponceau S provides a loading control. The experiment was performed three times with similar results.
(TIF)

S17 Fig The NB subdomain is sufficient for interaction with the LRR domain of RPP1_NdA. An NB-ARC1-ARC2 construct only weakly binds the LRR domain, regardless of whether the epitope tag is located C-terminally (A) or N-terminally (B). Constructs were transiently expressed in *Nicotiana benthamiana* and samples were collected at 48 hours post-infiltration for co-immunoprecipitation using α-Flag agarose beads. Staining of RuBisCO with Ponceau S provides a loading control. Asterisks indicate non-specific bands. The experiments were performed two times with similar results.
(TIF)

S18 Fig The N-TIR and LRR domains of RPP1_NdA associate in an effector-independent manner. A non-autoactive N-TIR domain mutant (R104A F106A) associates with the LRR, and the interaction is not disrupted by the presence of ATR1. Constructs were transiently expressed in *N*. *benthamiana* and samples were collected at 48 hours post-infiltration (E = ATR1_Emoy2, C = ATR1_Cala2). Co-immunoprecipitations were performed using α-Flag agarose beads. The expression of ATR1:citrine was detected with an α-GFP antibody. Asterisks indicate non-specific bands. Staining of RuBisCO with Ponceau S provides a loading control. Experiments were performed three times with similar results.
(TIF)

S1 Table Primers used in this study. (DOCX)
