# Supplementary figures and images for: Multiple Domain Associations within the Arabidopsis Immune Receptor RPP1 Regulate the Activation of Programmed Cell Death
Source: PLoS Pathog. 2016 Jul 18;12(7):e1005769. doi: 10.1371/journal.ppat.1005769 (PMC4948778; doi:10.1371/journal.ppat.1005769)

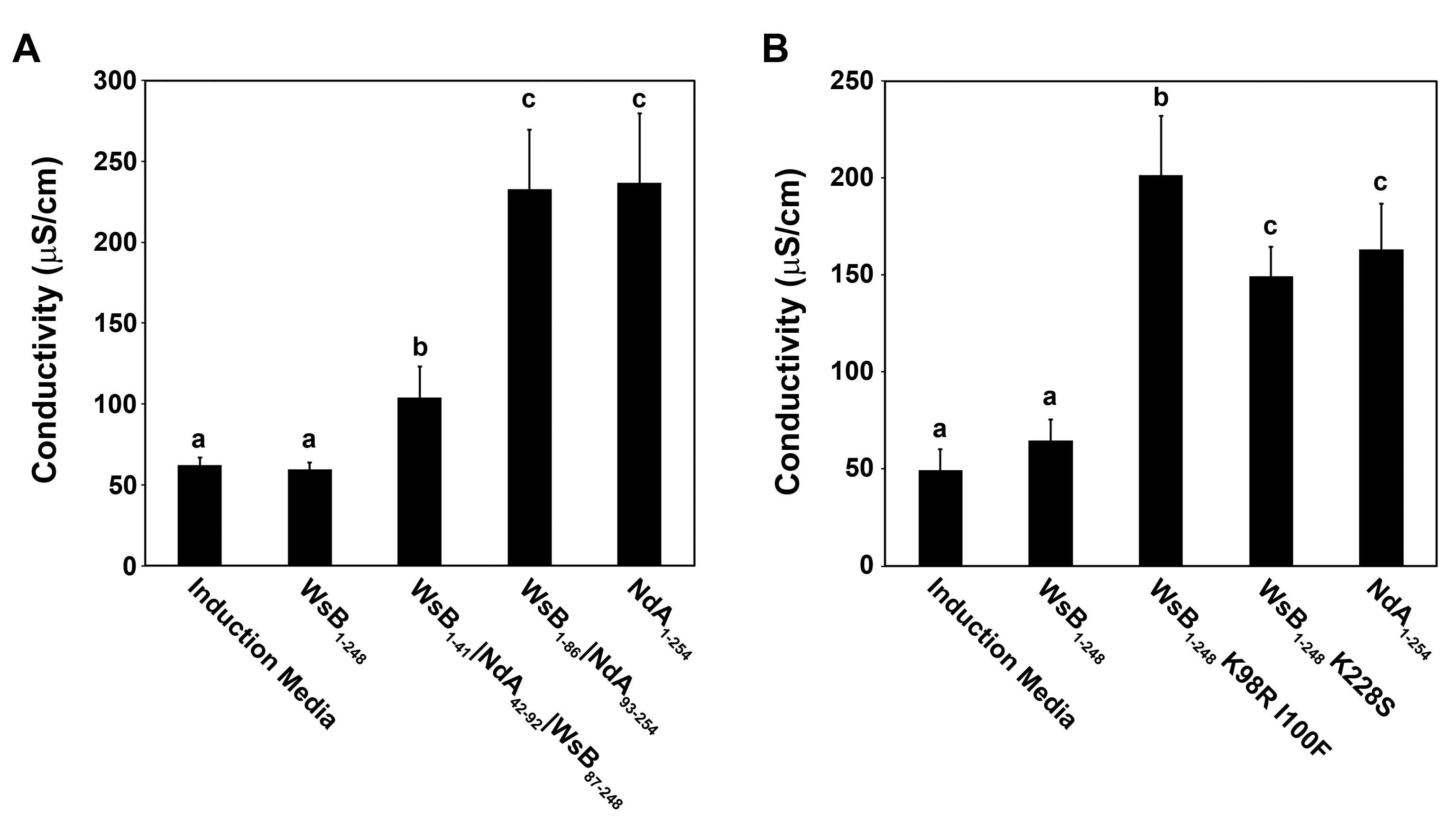

Supplement: S1 Fig — Data in (A) and (B) correspond to the constructs tested in (D) and (E) of Fig 1, respectively. The specific amino acids comprising each construct are indicated in subscript. Leaf discs representing approximately 4.5 cm2 of tissue were collected at approximately 28 hours post-infiltration and electrolyte concentration (conductivity) was measured 24 hours after collection. Error bars indicate standard deviation, and letters above data points indicate statistical significance groups as determined by pairwise Student’s t-tests (α = 0.05). Experiments were performed at least three times with similar results. (TIF) [file ppat.1005769.s001.tif]

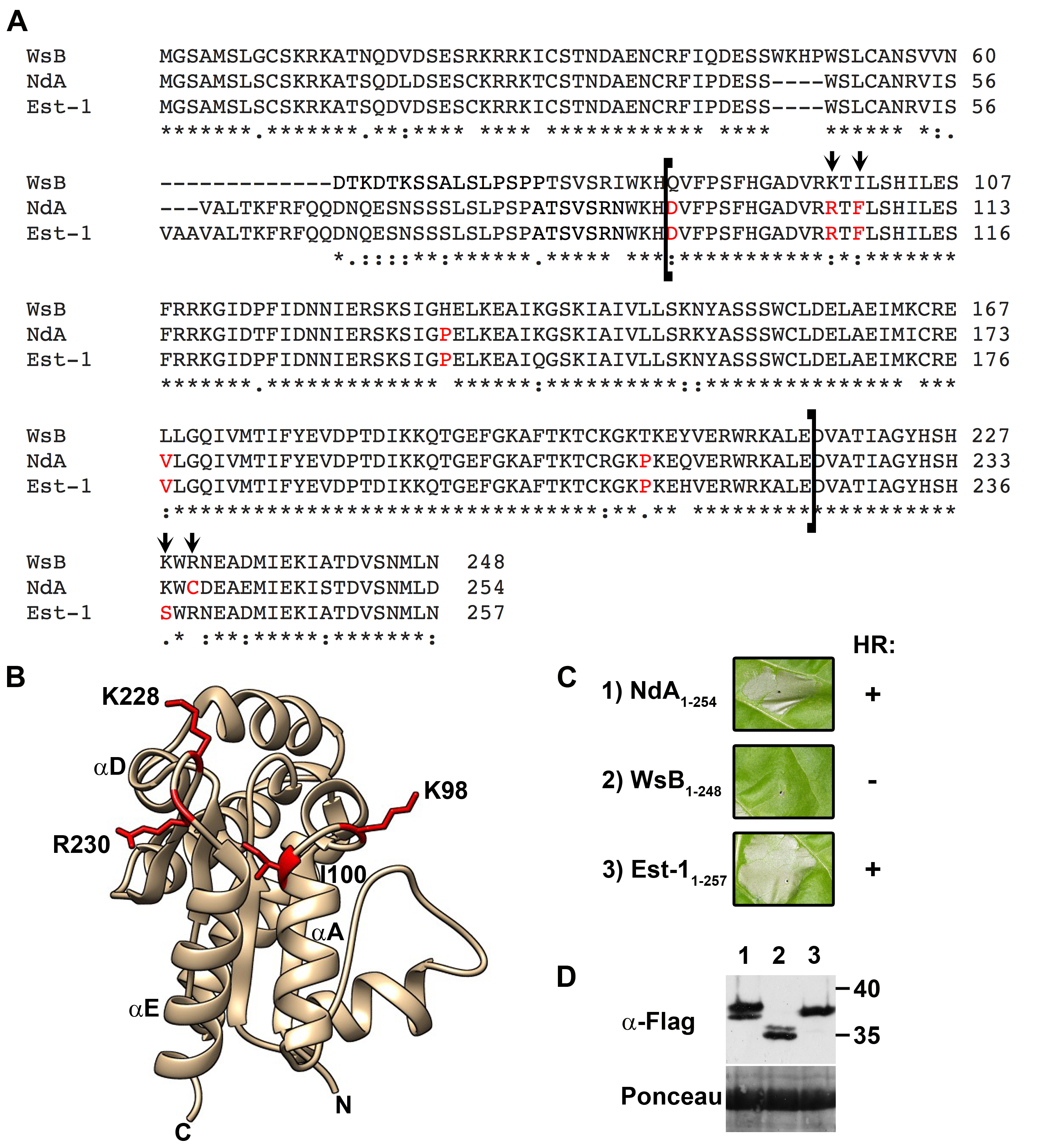

Supplement: S2 Fig — (A) Amino acid alignment of N-TIR domain sequences from the Niederzenz (NdA), Wassilewskija (WsB), and Estland-1 (Est-1) alleles of RPP1. The TIR domain borders predicted by Pfam are delimited by square brackets, and polymorphic amino acids with potential relevance to protein function are highlighted in red. Residues shown to influence autoactivity are indicated with arrowheads. (B) Functionally relevant polymorphic residues are also indicated on a putative structure of the RPP1_WsB TIR domain, derived by homology modeling using structural data from the L6 TIR domain (PDB: 3OZI). Relevant α-helices are also annotated. (C) Autoactivity of N-TIR domains from different RPP1 alleles. Constructs were tested in Nicotiana tabacum via Agrobacterium-mediated transient expression and images of hypersensitive response (HR) phenotypes were captured at 48 hours post-infiltration. The presence or absence of HR is indicated by a “+” or “-“, respectively. The specific amino acids comprising each construct are indicated in subscript. (D) An α-Flag antibody was used to evaluate protein expression, while staining of RuBisCO with Ponceau S provided a loading control. The experiment was performed three times with similar results. (TIF) [file ppat.1005769.s002.tif]

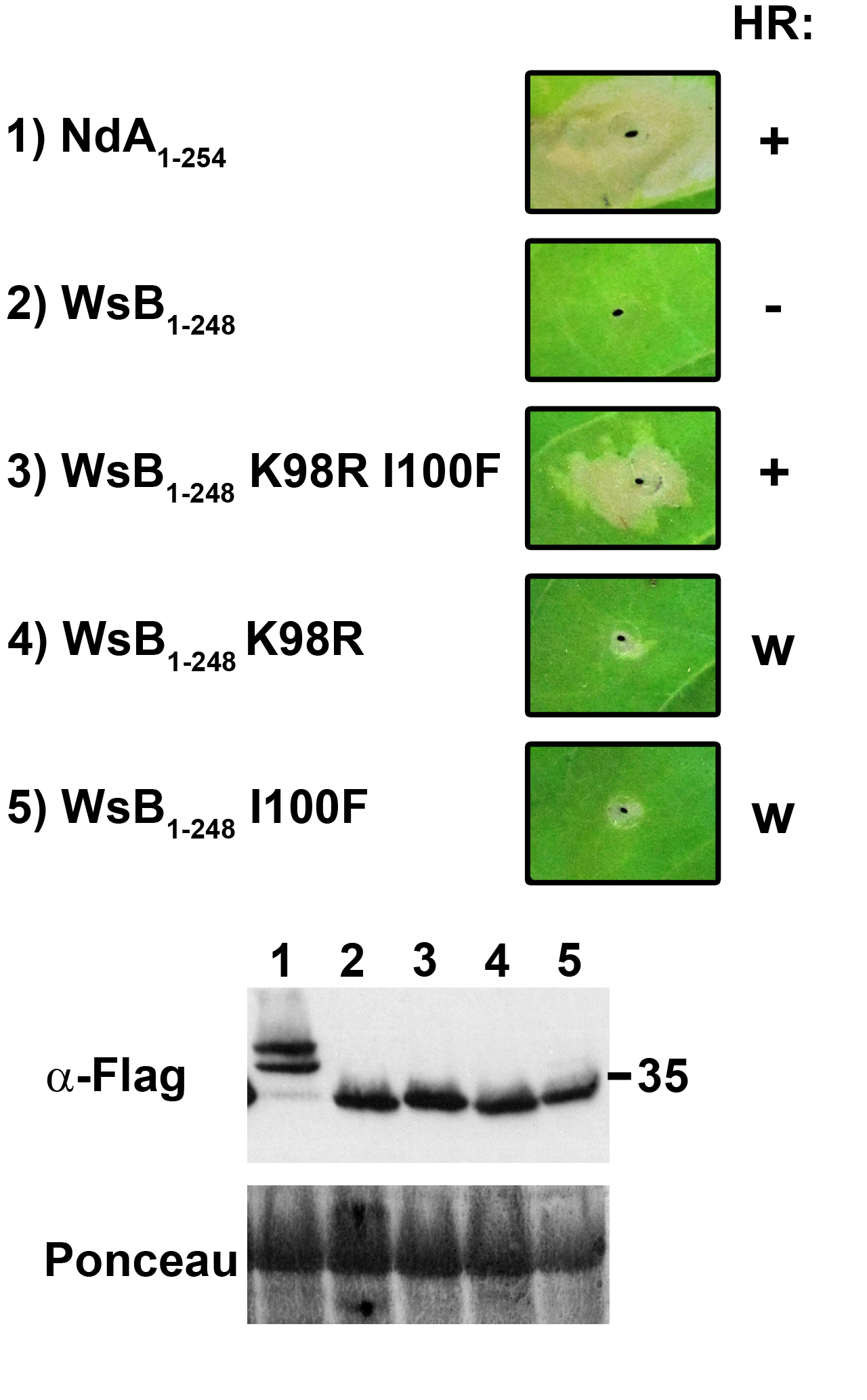

Supplement: S3 Fig — Constructs were tested in Nicotiana tabacum via Agrobacterium-mediated transient expression and images of hypersensitive response (HR) phenotypes were captured at 48 hours post-infiltration. HR phenotypes are scored as negative (-), weak (w), or strong (+). The specific amino acids comprising each construct are indicated in subscript. An α-Flag antibody was used to evaluate protein expression, while staining of RuBisCO with Ponceau S provided a loading control. The experiment was performed three times with similar results. (TIF) [file ppat.1005769.s003.tif]

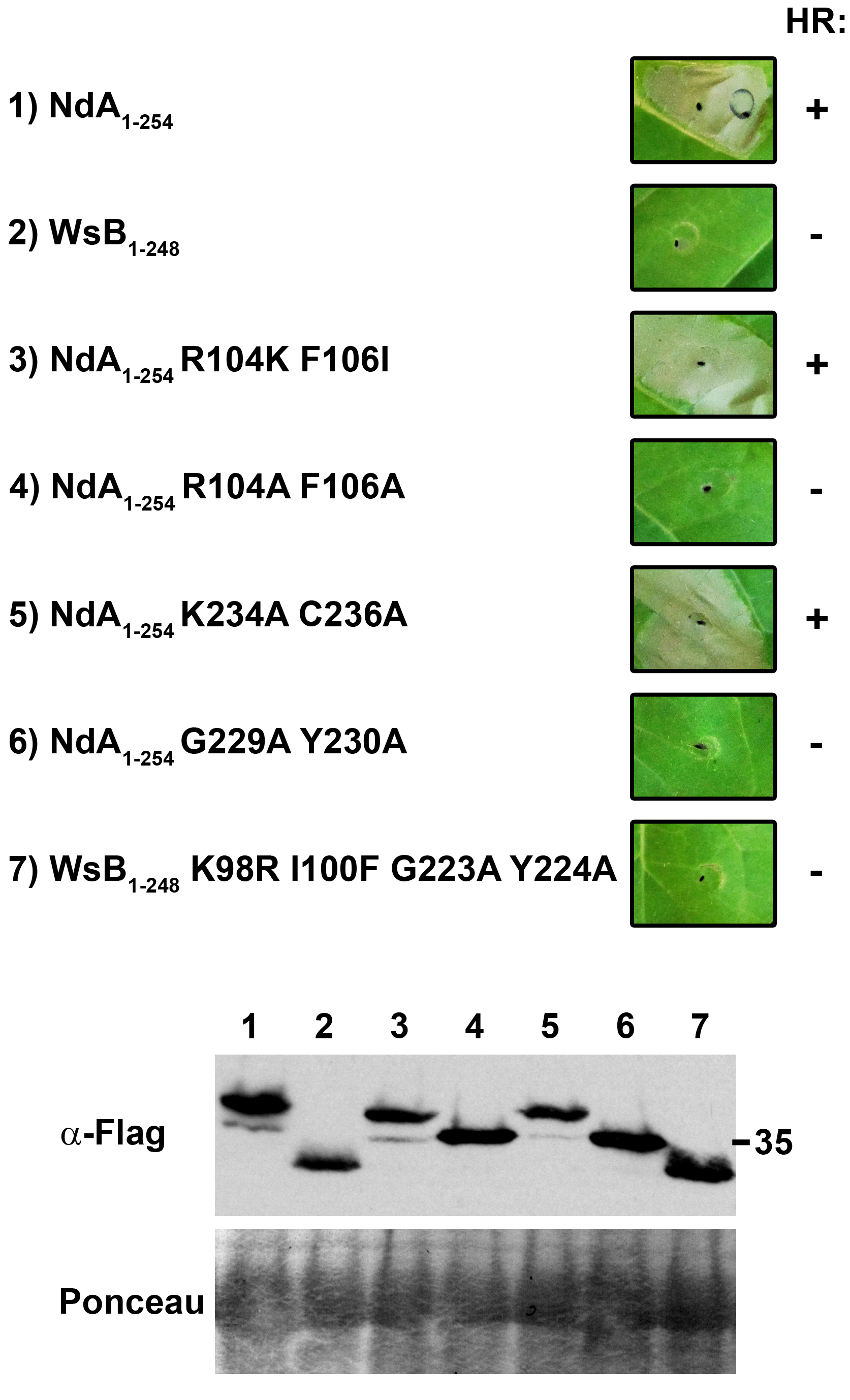

Supplement: S4 Fig — Constructs were tested in Nicotiana tabacum via Agrobacterium-mediated transient expression and images of hypersensitive response (HR) phenotypes were captured at 48 hours post-infiltration. The presence or absence of HR is indicated by a “+” or “-“, respectively. The specific amino acids comprising each construct are indicated in subscript. An α-Flag antibody was used to evaluate protein expression, while staining of RuBisCO with Ponceau S provided a loading control. The experiment was performed three times with similar results. (TIF) [file ppat.1005769.s004.tif]

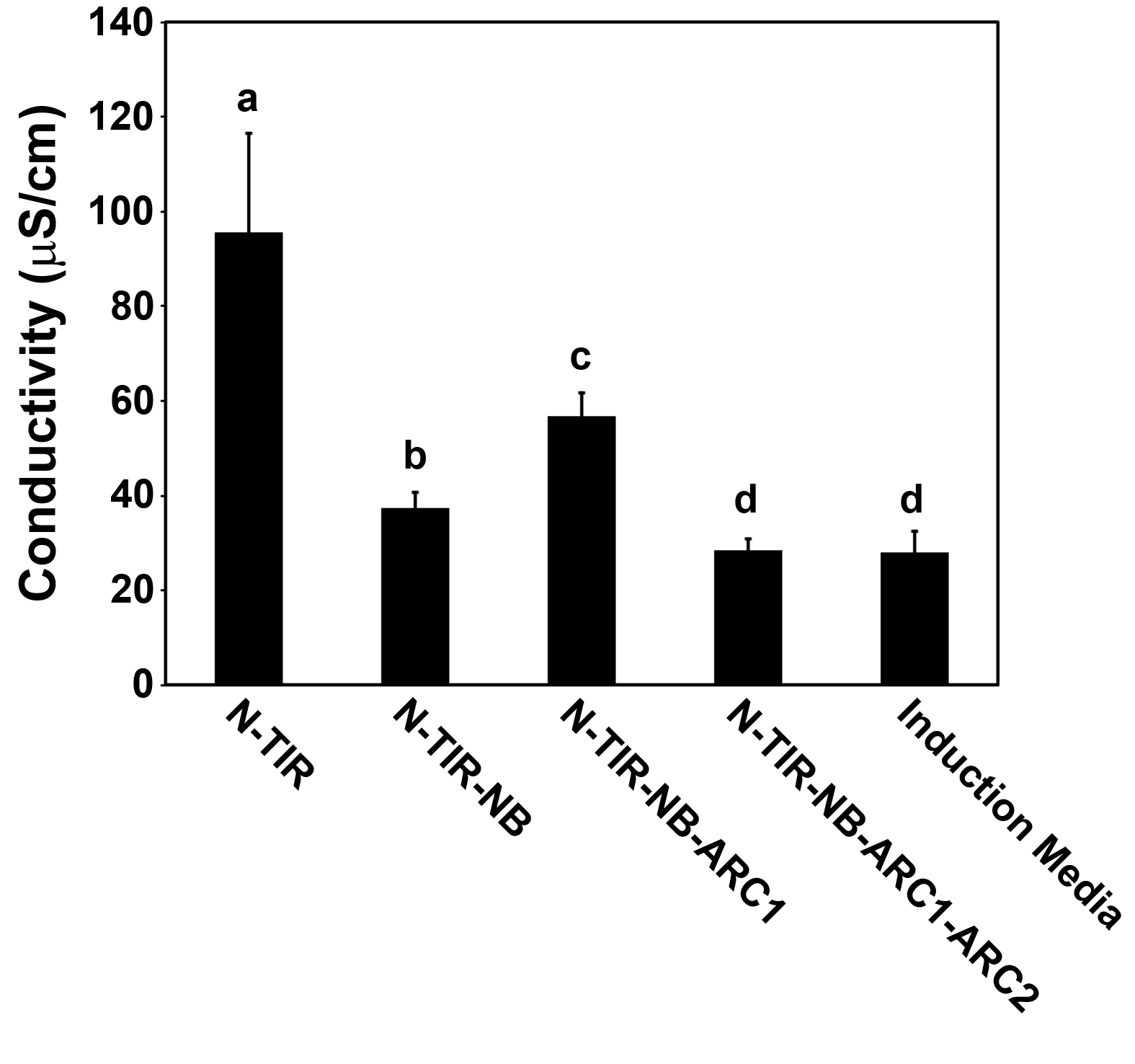

Supplement: S5 Fig — Leaf discs representing approximately 4.5 cm2 of tissue were collected at approximately 28 hours post-infiltration and electrolyte concentration (conductivity) was measured 24 hours after collection. Error bars indicate standard deviation, and letters above data points indicate statistical significance groups as determined by pairwise Student’s t-tests (α = 0.05). The experiment was performed three times with similar results. (TIF) [file ppat.1005769.s005.tif]

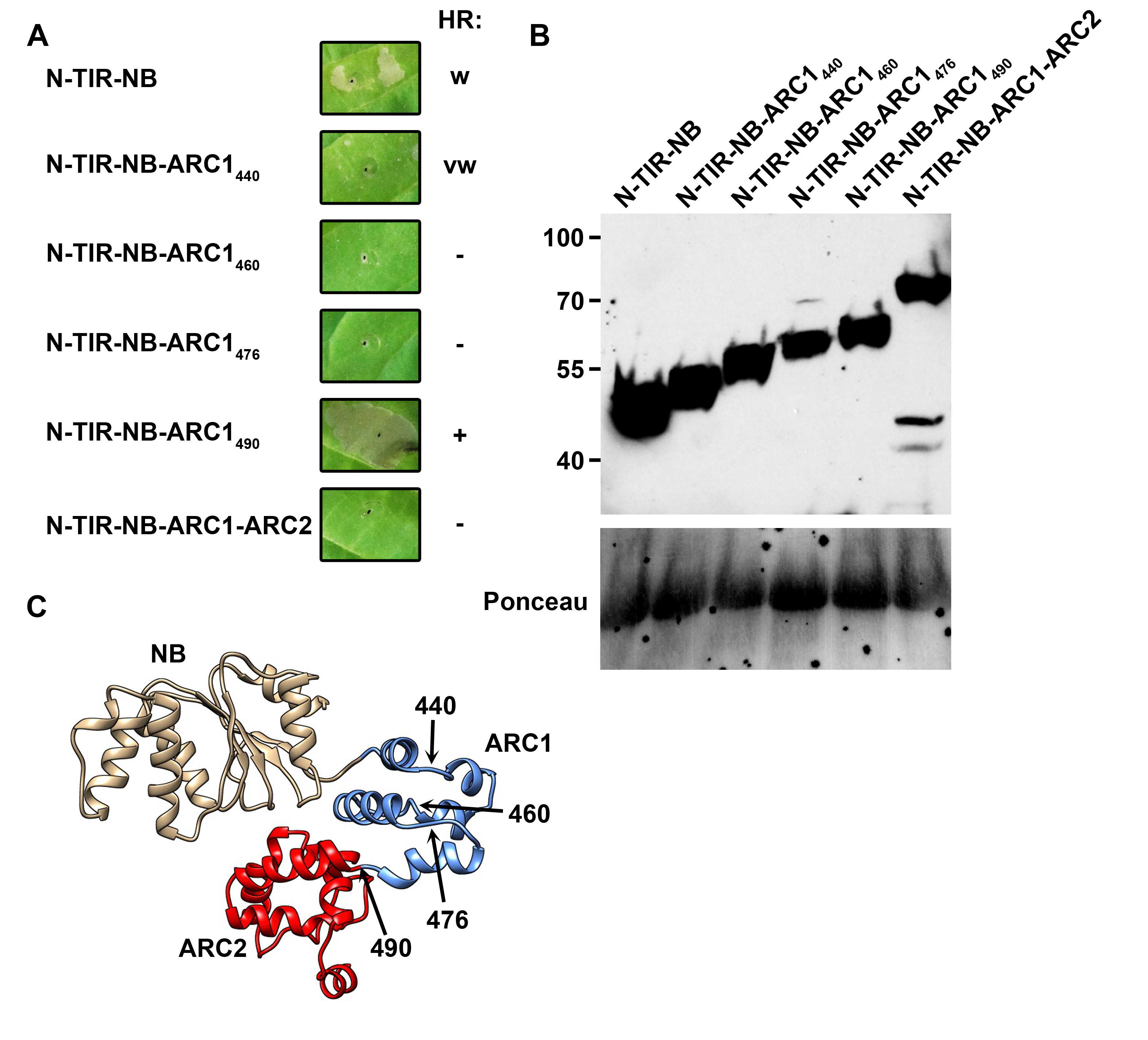

Supplement: S6 Fig — (A) Hypersensitive response (HR) phenotypes associated with C-terminal truncations of an N-TIR-NB-ARC1 construct from the NdA allele. The amino acids at the C-terminus of each truncation are indicated in subscript. Constructs were tested in Nicotiana tabacum via Agrobacterium-mediated transient expression and images were captured at 48 hours post-infiltration. HR phenotypes are scored as negative (-), very weak (vw), weak (w), or strong (+). (B) An α-Flag antibody was used to evaluate protein expression, while staining of RuBisCO with Ponceau S provided a loading control. Experiments were performed at least three times with similar results. (C) Predicted structure of the RPP1 NB-ARC domain based on homology modeling using the Drosophila Dark protein (PDB:4v4l) as a template. The three subdomains of this region are highlighted and the locations of each truncation tested in (A) are indicated by arrows. (TIF) [file ppat.1005769.s006.tif]

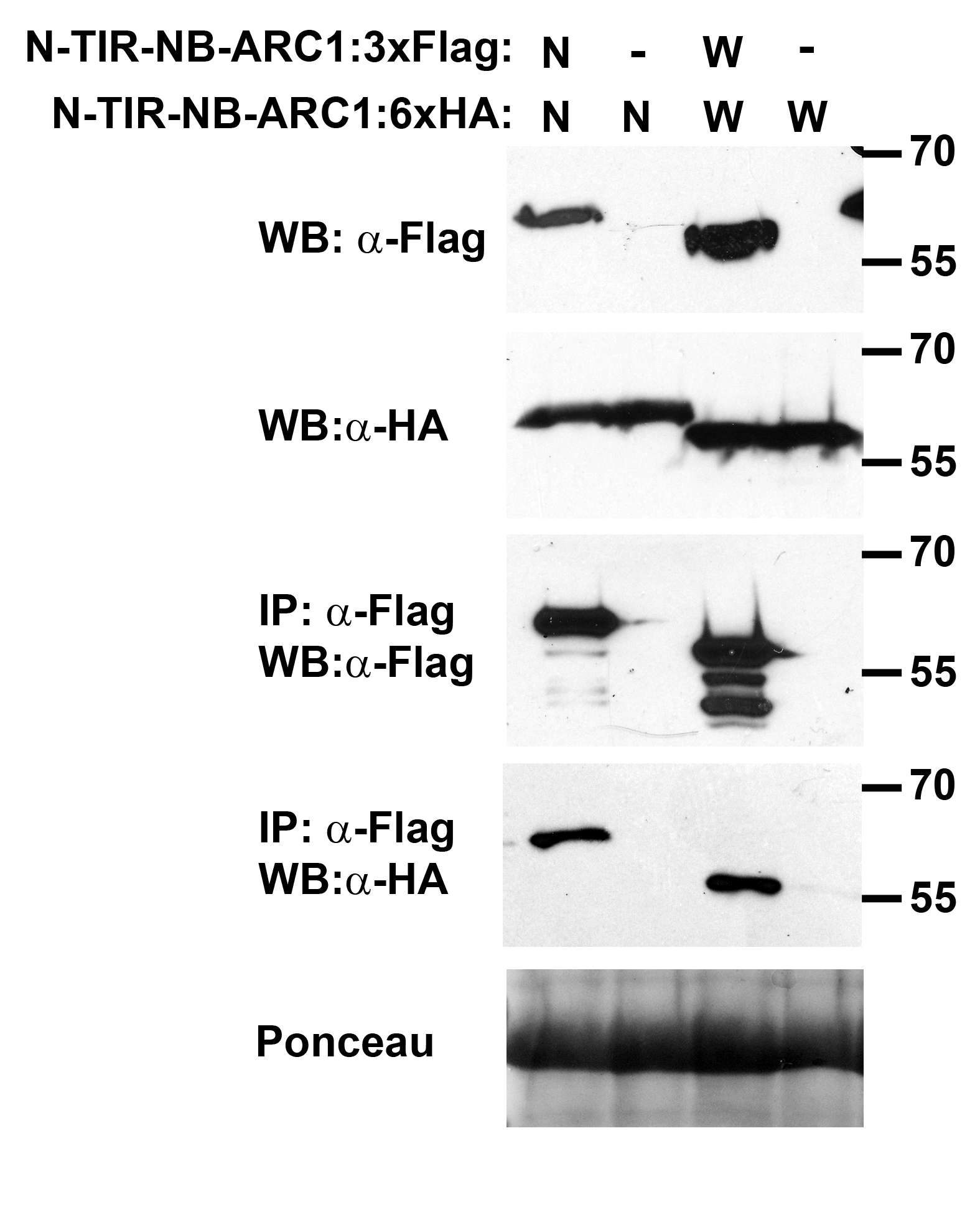

Supplement: S7 Fig — Differentially epitope-tagged N-TIR-NB-ARC1 proteins from the Niederzenz (N) and Wassilewskija (W) alleles of RPP1 were transiently expressed in Nicotiana benthamiana and samples were collected at 48 hours post-infiltration for co-immunoprecipitation using α-Flag agarose beads. Staining of RuBisCO with Ponceau S provides a loading control. The experiment was performed three times with similar results. (TIF) [file ppat.1005769.s007.tif]

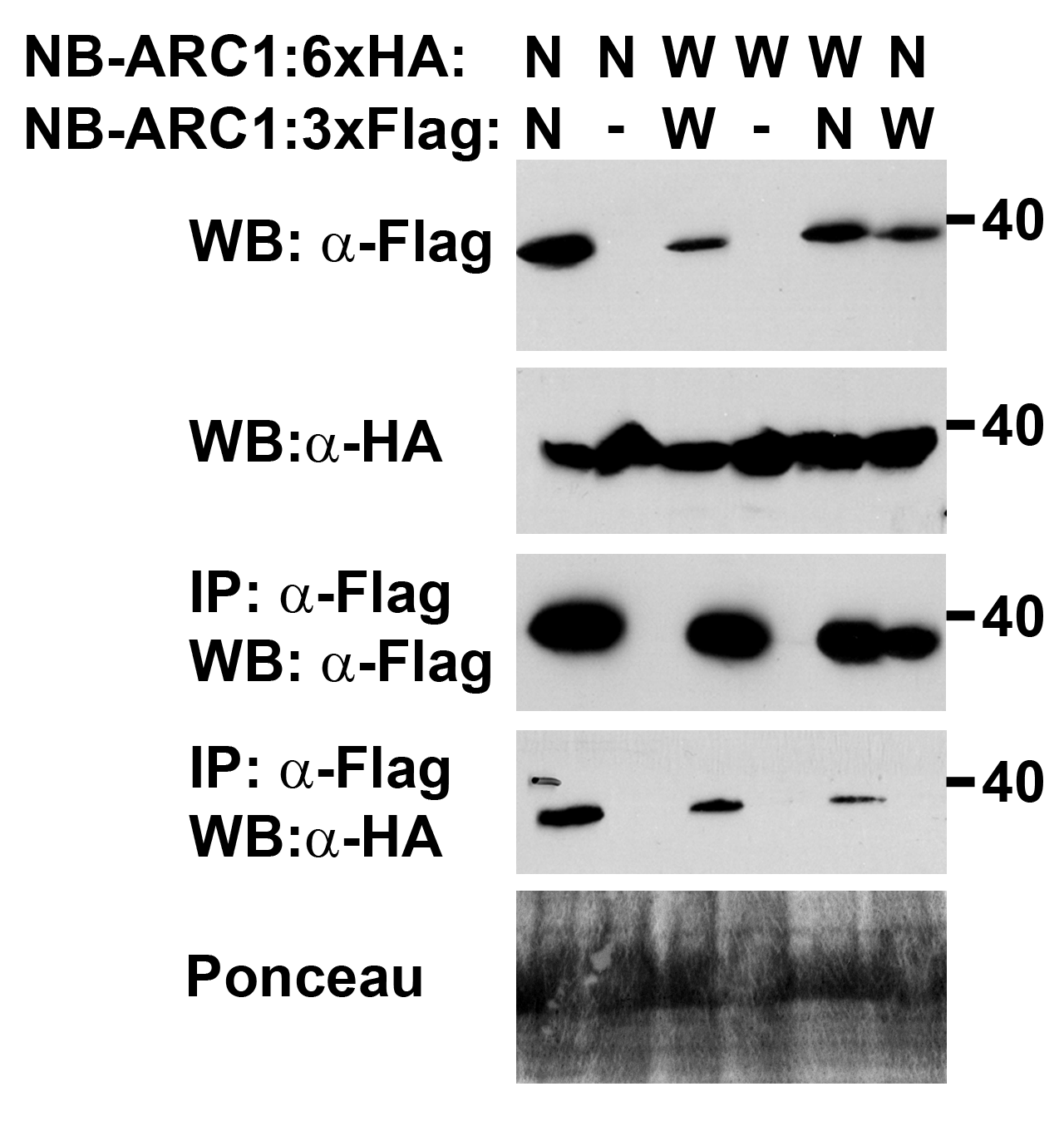

Supplement: S8 Fig — Constructs were transiently expressed in Nicotiana benthamiana and samples were collected at 48 hours post-infiltration for co-immunoprecipitation using α-Flag agarose beads. Staining of RuBisCO with Ponceau S provides a loading control. The experiment was performed three times with similar results. (TIF) [file ppat.1005769.s008.tif]

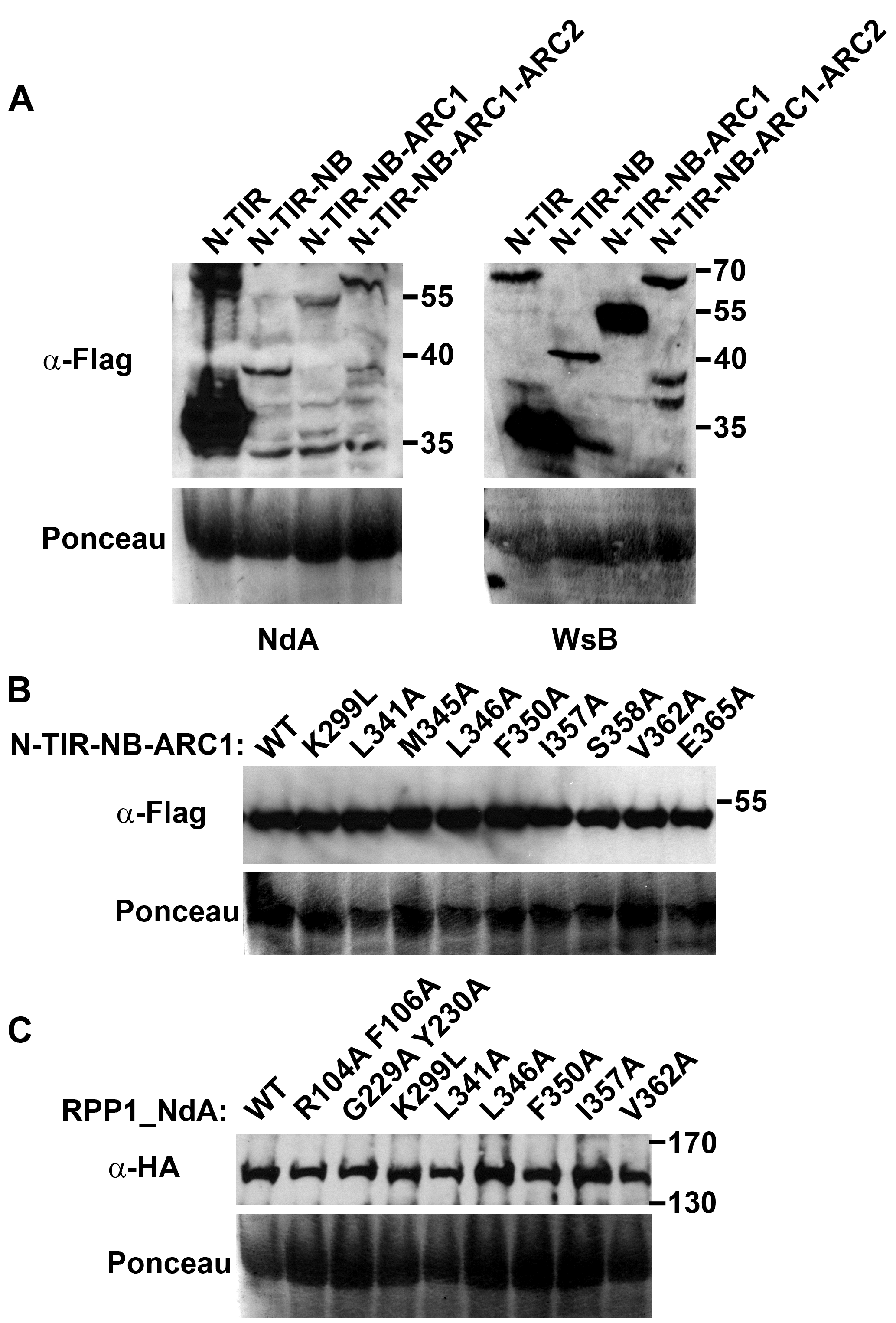

Supplement: S9 Fig — (A) N-TIR domain addition constructs tested in Fig 3A, including sequences from the RPP1 alleles Niederzenz (NdA) and Wassilewskija (WsB). (B) N-TIR-NB-ARC1 (NdA) constructs tested in Fig 3C. (C) Full-length RPP1 (NdA) constructs tested in Fig 4. Constructs were transiently expressed in Nicotiana benthamiana and samples were collected at 24 hours post-infiltration (hpi) for (A) and (B) or 48 hpi for (C). Staining of RuBisCO with Ponceau S provides a loading control. The experiments were performed three times with similar results. (TIF) [file ppat.1005769.s009.tif]

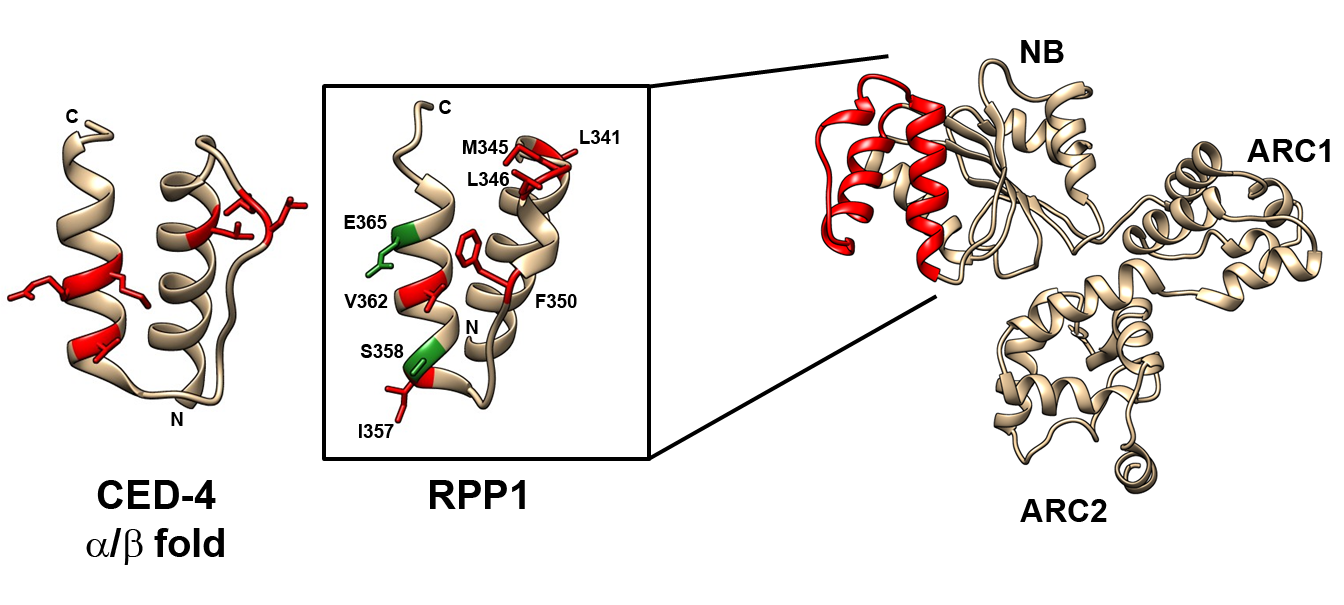

Supplement: S10 Fig — The α-helices that comprise the oligomerization interface of the Caenorhabditis elegans CED-4 protein (PDB: 2A5Y) are shown on the left, with residues that contribute to oligomerization highlighted in red. The corresponding region of RPP1 is shown at center; residues required for induction of the hypersensitive response are highlighted in red, while those that were dispensable for this response are depicted in green. The predicted structure of the RPP1 NB-ARC domain is shown on the right and is derived from homology modeling using the Drosophila Dark protein (PDB:4v4l) as a template. The putative oligomerization interface region is highlighted in red. Note that the enlarged image of this region is rotated approximately 90° counterclockwise on the y-axis for improved visibility of the residues of interest. (TIF) [file ppat.1005769.s010.tif]

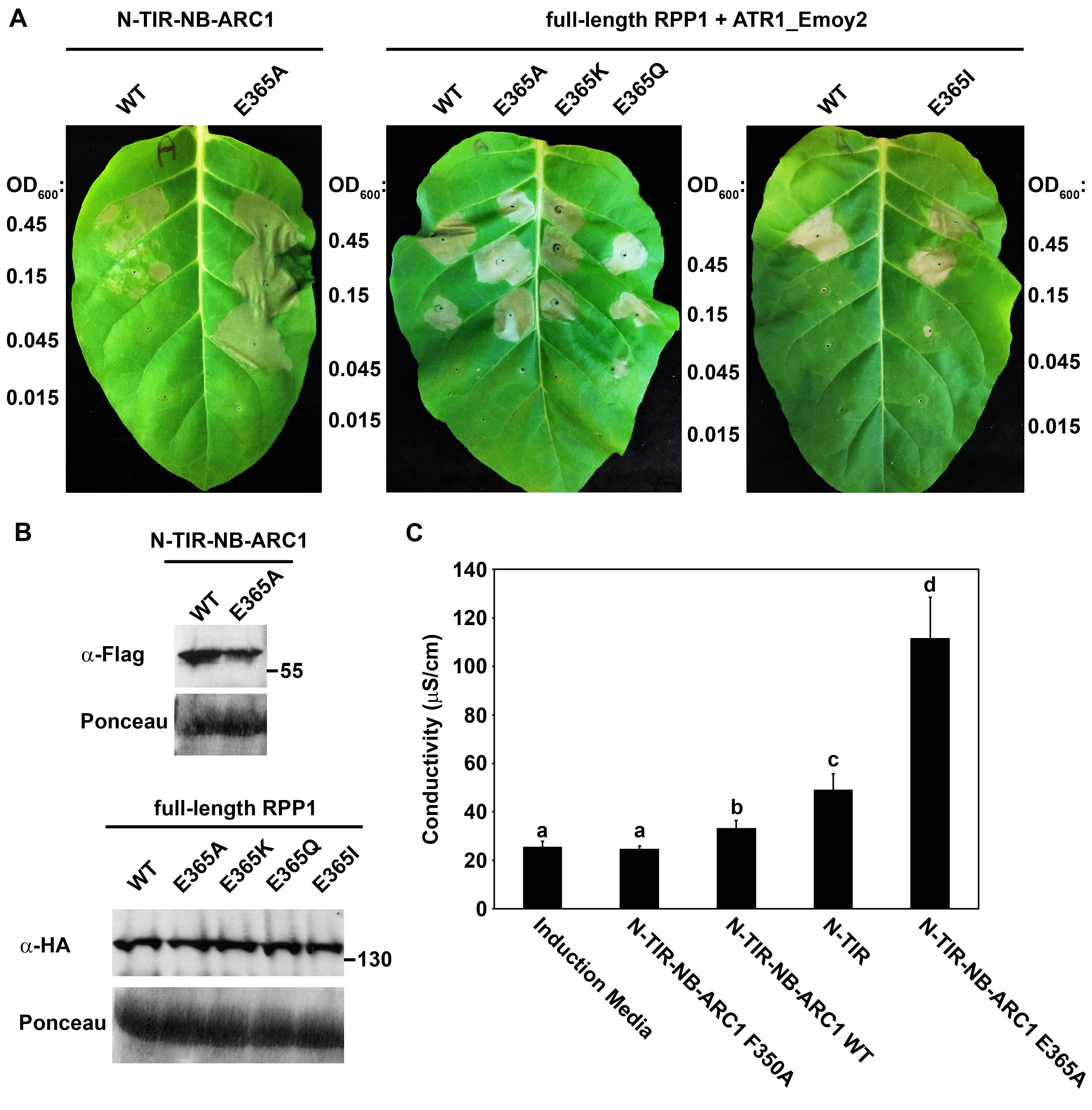

Supplement: S11 Fig — (A) Hypersensitive response phenotypes elicited by N-TIR-NB-ARC1 constructs or by co-expression of full-length RPP1_NdA and ATR1_Emoy2. Constructs were tested in Nicotiana tabacum via Agrobacterium-mediated transient expression, using a range of inoculum concentrations (as measured by OD600) to allow a comparison of the strength of cell death induction by wild-type (WT) and E365 substitution mutants. Images were captured at 48 hours post-infiltration (hpi). (B) An α-Flag antibody was used to evaluate protein expression, while staining of RuBisCO with Ponceau S provided a loading control. (C) Quantification of electrolyte leakage induced by various N-TIR-NB-ARC1 constructs. Leaf discs representing approximately 4.5 cm2 of tissue were collected at approximately 28 hpi and electrolyte concentration (conductivity) was measured 24 hours after collection. The non-autoactive F350A mutant and autoactive N-TIR domain were included as negative and positive controls, respectively. Error bars indicate standard deviation, and letters above data points indicate statistical significance groups as determined by pairwise Student’s t-tests (α = 0.05). Experiments were performed three times with similar results. (TIF) [file ppat.1005769.s011.tif]

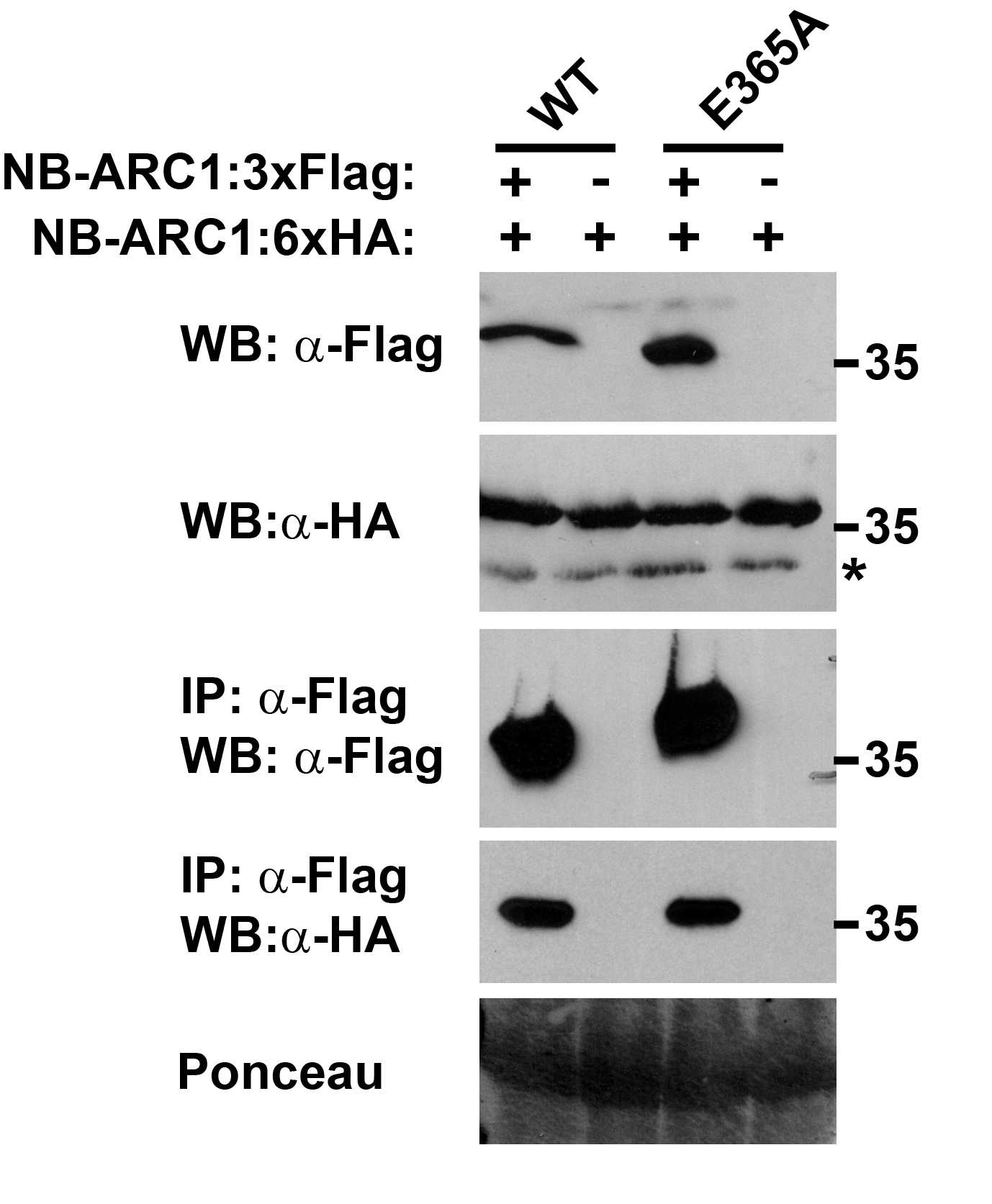

Supplement: S12 Fig — Constructs were transiently expressed in Nicotiana benthamiana and samples were collected at 48 hours post-infiltration for co-immunoprecipitation using α-Flag agarose beads. Asterisks indicate non-specific bands. Staining of RuBisCO with Ponceau S provides a loading control. The experiment was performed three times with similar results. (TIF) [file ppat.1005769.s012.tif]

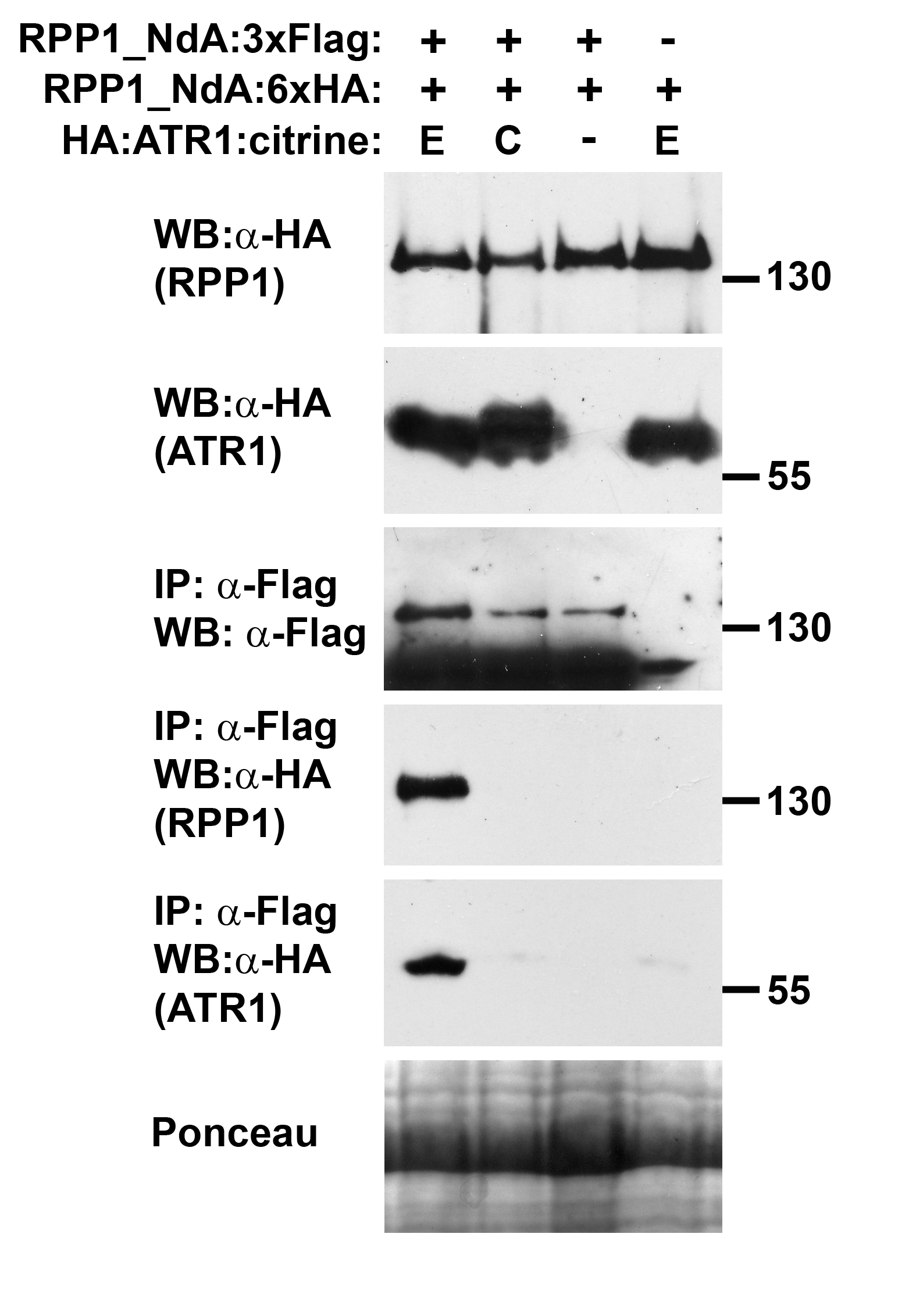

Supplement: S13 Fig — Differentially epitope-tagged RPP1_NdA proteins were transiently co-expressed with either ATR1_Emoy2 (E) or ATR1_Cala2 (C) in Nicotiana benthamiana and samples were collected at 36 hours post-infiltration for co-immunoprecipitation using α-Flag agarose beads. Due to a non-specific signal at the expected molecular weight of ATR1:citrine when the elution fraction was probed with an α-GFP antibody, HA:ATR1:citrine constructs were used and ATR1 expression detected with an α-HA antibody. Staining of RuBisCO with Ponceau S provides a loading control. The experiment was performed three times with similar results. (TIF) [file ppat.1005769.s013.tif]

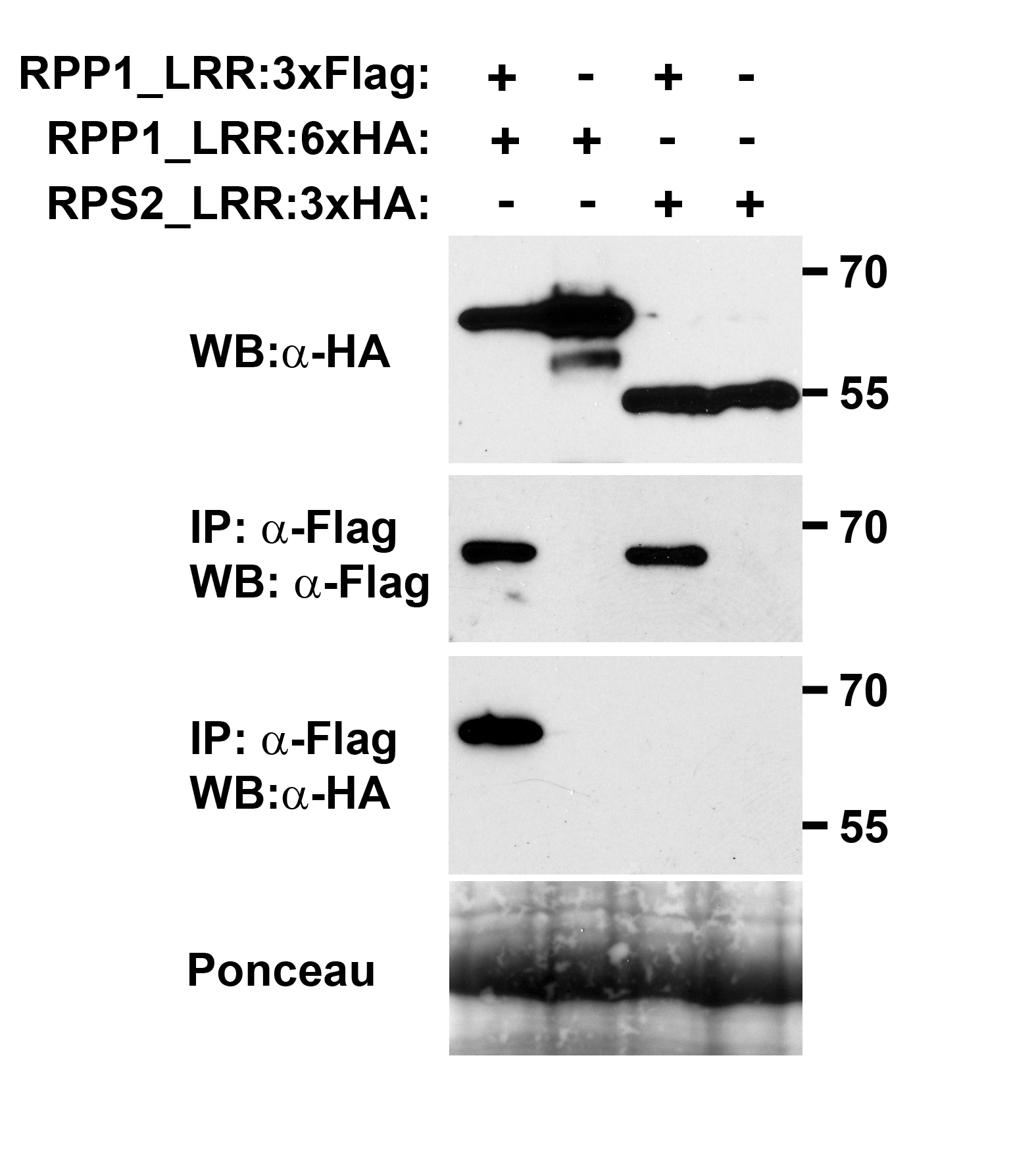

Supplement: S14 Fig — While self-association of the RPP1_NdA LRR is detected, this protein does not interact with the LRR domain of RPS2. Constructs were transiently expressed in Nicotiana benthamiana and samples were collected at 48 hours post-infiltration for co-immunoprecipitation using α-Flag agarose beads. Staining of RuBisCO with Ponceau S provides a loading control. Experiments were performed three times with similar results. (TIF) [file ppat.1005769.s014.tif]

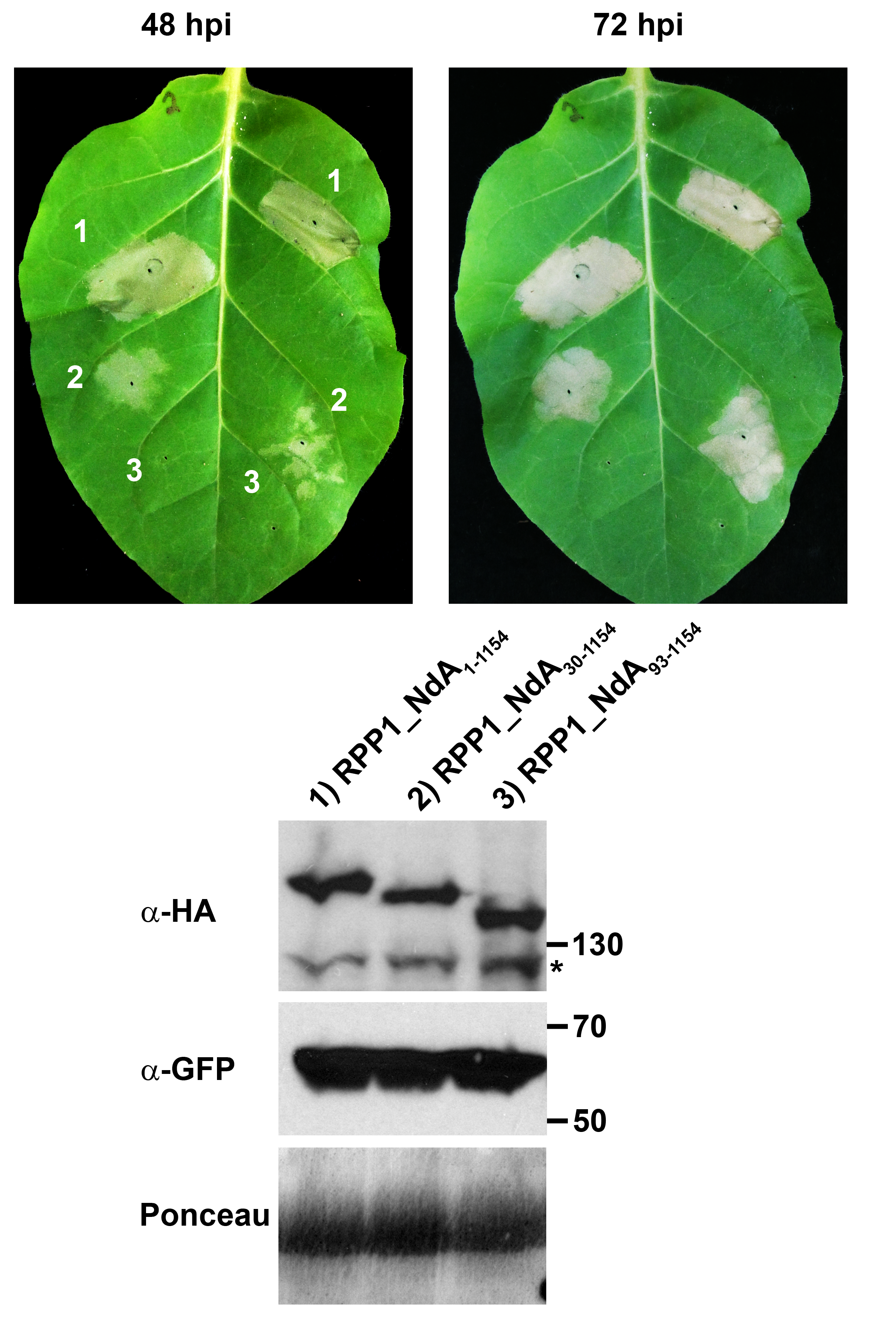

Supplement: S15 Fig — Constructs were tested in Nicotiana tabacum via Agrobacterium-mediated transient co-expression with ATR1_Emoy2, and images of hypersensitive response phenotypes were captured at 48 and 72 hours post-infiltration (hpi). The constructs included RPP1_NdA1-1154 (1), RPP1_NdA30-1154 (2), and RPP1_NdA93-1154 (3), where the specific amino acids comprising each construct are indicated in subscript. An α-HA antibody was used to evaluate protein expression, and the expression of ATR1_Emoy2:citrine was detected with an α-GFP antibody. Staining of RuBisCO with Ponceau S provided a loading control. The experiment was performed three times with similar results. (TIF) [file ppat.1005769.s015.tif]

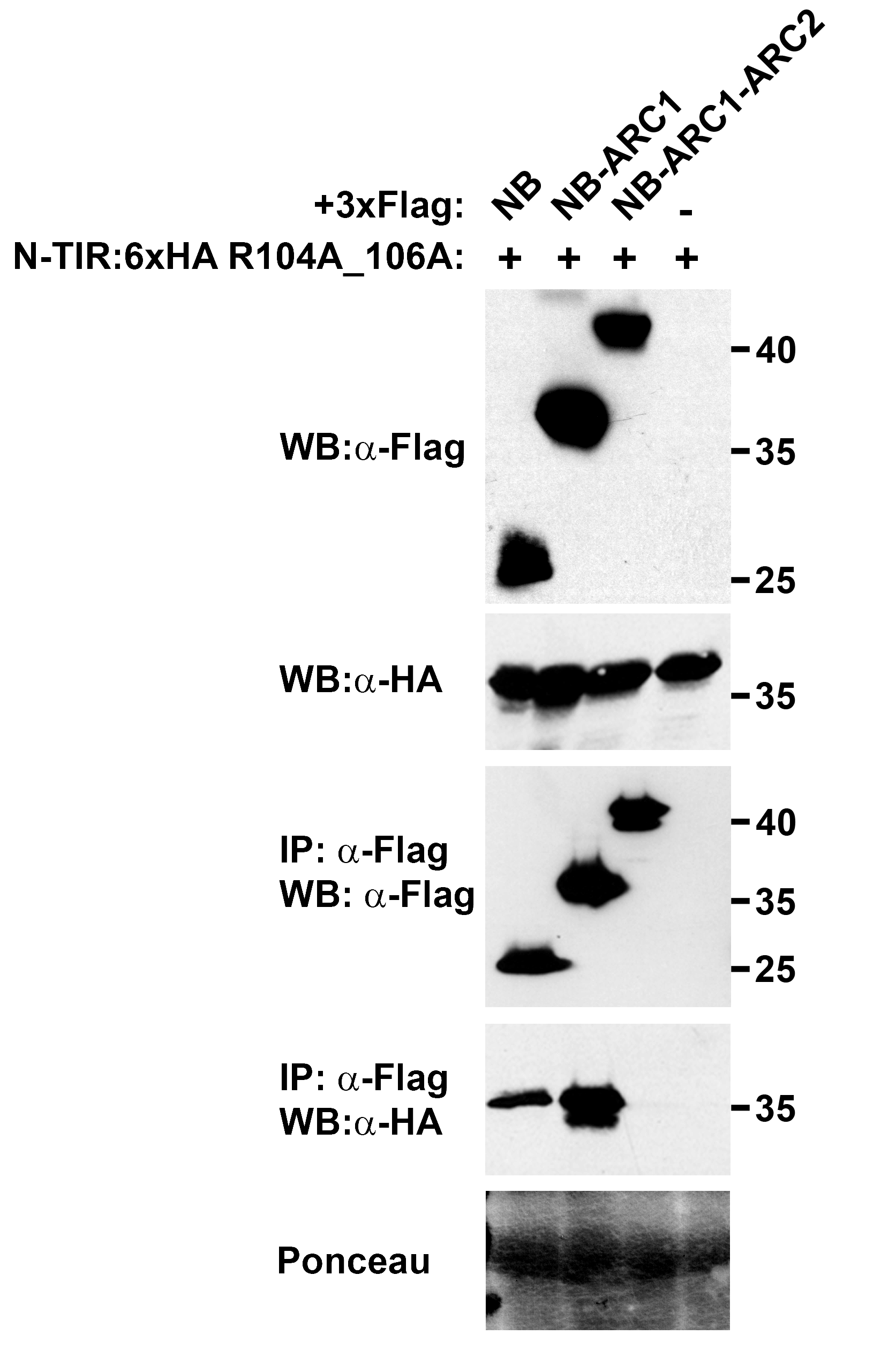

Supplement: S16 Fig — Constructs were transiently expressed in Nicotiana benthamiana and samples were collected at 48 hours post-infiltration for co-immunoprecipitation using α-Flag agarose beads. Staining of RuBisCO with Ponceau S provides a loading control. The experiment was performed three times with similar results. (TIF) [file ppat.1005769.s016.tif]

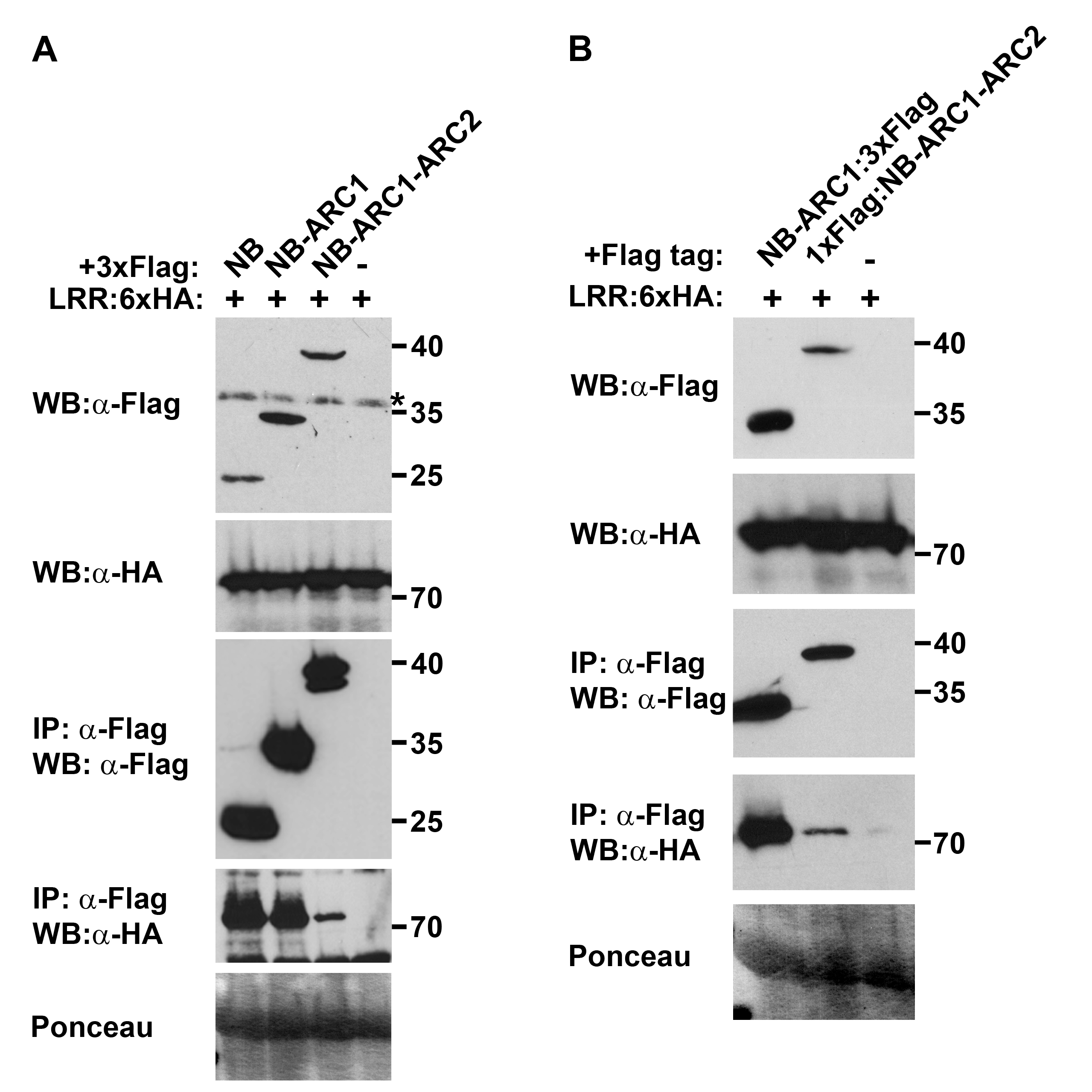

Supplement: S17 Fig — An NB-ARC1-ARC2 construct only weakly binds the LRR domain, regardless of whether the epitope tag is located C-terminally (A) or N-terminally (B). Constructs were transiently expressed in Nicotiana benthamiana and samples were collected at 48 hours post-infiltration for co-immunoprecipitation using α-Flag agarose beads. Staining of RuBisCO with Ponceau S provides a loading control. Asterisks indicate non-specific bands. The experiments were performed two times with similar results. (TIF) [file ppat.1005769.s017.tif]

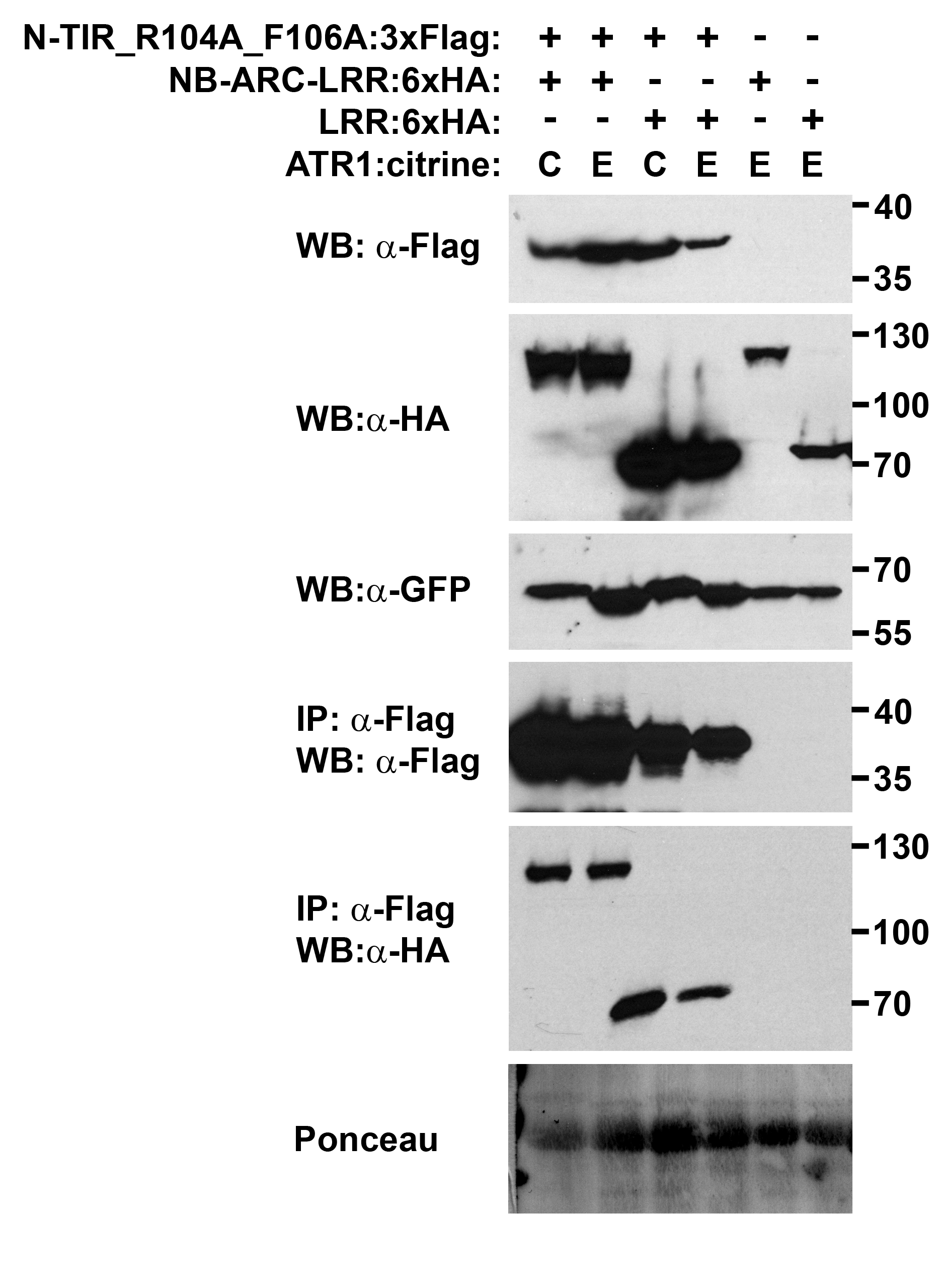

Supplement: S18 Fig — A non-autoactive N-TIR domain mutant (R104A F106A) associates with the LRR, and the interaction is not disrupted by the presence of ATR1. Constructs were transiently expressed in N. benthamiana and samples were collected at 48 hours post-infiltration (E = ATR1_Emoy2, C = ATR1_Cala2). Co-immunoprecipitations were performed using α-Flag agarose beads. The expression of ATR1:citrine was detected with an α-GFP antibody. Asterisks indicate non-specific bands. Staining of RuBisCO with Ponceau S provides a loading control. Experiments were performed three times with similar results. (TIF) [file ppat.1005769.s018.tif]
