# Supplementary material for: Multiple Domain Associations within the Arabidopsis Immune Receptor RPP1 Regulate the Activation of Programmed Cell Death
Source: PLoS Pathog. 2016 Jul 18;12(7):e1005769. doi: 10.1371/journal.ppat.1005769 (PMC4948778; doi:10.1371/journal.ppat.1005769)
Supplement: S1 Table — (DOCX) [file ppat.1005769.s019.docx]

**Table S1. Primers used in this study**

| **Application** | **Primer Name** | **Sequence (5' to 3')** |  |
| --- | --- | --- | --- |
| **RPP1 Domain Constructs** |  |  |  |
|  | NotI_aa1_F | ATTGCGGCCGCcATGGGTTCTGCAATGAGCTTG |  |
|  | NotI_NdA_aa30_F | ATTGCGGCCGCcatgTGCTCAACAAACGATGCTGAG |  |
|  | NotI_NdA_aa93_F | ATTGCGGCCGCcatgGTCTTCCCGAGCTTCCACGGAG |  |
|  | NotI_NdA_aa255_F | ATTGCGGCCGCcatgTTATCCATACCATCAAAAG |  |
|  | NotI_WsB_aa249_F | ATTGCGGCCGCcatgAGTTTCAAACCATCAAGAG |  |
|  | NotI_NdA_aa606_F | ATTGCGGCCGCcatgGATAGTAGATGTTTTATAG |  |
|  | SpeI_NdA_aa233ns_R | ggactagtGTGAGAATGATATCCGGCGATTG |  |
|  | XbaI_NdA_aa254ns_R | gctctagaATCCAACATGTTTGAAACATCAG |  |
|  | XbaI_WsB_aa248ns_R | gctctagaATTTAACATGTTTGAAACATCAGTG |  |
|  | XbaI_NdA_aa272_R | GCTCTAGACATATGAGCTGCCATCCCAACAAAATCATC |  |
|  | SpeI_WsB_aa418ns_R | ggactagtATATCCCACTTTGTAAACATGATTG |  |
|  | SpeI_NdA_aa424ns_R | ggactagtAGATTTCACTTTGTAAACATGATTG |  |
|  | XbaI_NdA_aa440_R | gctctagaACCAAAGGCATTCATACAGAAG |  |
|  | XbaI_NdA_aa460_R | gctctagaACCAGCAAGGCAGGTAACTTC |  |
|  | XbaI_NdA_aa476_R | gctctagaCATTCCCCGCAGAGCCGAGCCT |  |
|  | XbaI_NdA_aa490_R | gctctagaAGTTTTTAACCTTGGTAGTGTTC |  |
|  | XbaI_NdA_aa597_R | GCTCTAGAACATATATCCCTTTCACCAAC |  |
|  | XbaI_WsB_aa590_R | GCTCTAGAACATATATCCCTTTCACCAAC |  |
|  | XbaI_NdA_aa1154_R | gctctagaTCTTTGGAGTATCCCGCATTC |  |
|  | SpeI_WsB1221_R | GGACTAGTACAGTGGTCGCAGCTAGAAAC |  |
| **RPP1 Chimera**  **Constructs** | |  | |
|  | CO_aa42_WsB_F | CTGAGAATTGCAGATTCATTCAGGATGAGTCATCTTGGAAAC |  |
|  | CO_aa42_NdA_F | CTGAGAATTGCAGATTCATTCCGGATGAGTCATCTTGGAG |  |
|  | Univ_aa41_R | AATGAATCTGCAATTCTCAG |  |
|  | F-TIRCO-Uni | GTCTTCCCGAGCTTCCA |  |
|  | R-TIRCO-5'WsB | TGGAAGCTCGGGAAGACTTGGTGTTTCCAGATTCGAG |  |
|  | R-TIRCO-5'NdA | TGGAAGCTCGGGAAGACATCGTGTTTCCAGTTTCGAG |  |
| **TIR Expression**  **Constructs** | |  | |
|  | LIC_N1_F | TACTTCCAATCCAATGCA GGT TCT GCA ATG AGCTTGAG |  |
|  | LIC_WsB84_F | TACTTCCAATCCAATGCAAAACACCAAGTCTTCCCGAG |  |
|  | LIC_WsB248_R | TTATCCACTTCCAATGTTATTAATTTAACATGTTTGAAACATCAGTG |  |
|  | LIC_N90_F | TACTTCCAATCCAATGCAAAACACGATGTCTTCCCGAGCTTCCAC |  |
|  | LIC_N254_R | TTATCCACTTCCAATGTTATTAATCCAACATGTTTGAAACATCAG |  |
| **Site-Directed Mutagenesis** |  |  |  |
|  | WsB_K98R_I100F_F | GCAGATGTTCGAAGAACCTTTCTTAGTCACATC |  |
|  | WsB_K98R_I100F_R | GATGTGACTAAGAAAGGTTCTTCGAACATCTGC |  |
|  | NdA_R104A_F106A_F | CACGGAGCAGATGTCCGAGCAACCGCTCTTAGTCACATCCTCG |  |
|  | NdA_R104A_F106A_R | CGAGGATGTGACTAAGAGCGGTTGCTCGGACATCTGCTCCGTG |  |
|  | WsB_K98R_F | GCAGATGTTCGAAGAACCATTCTTAGTC |  |
|  | WsB_K98R_R | GACTAAGAATGGTTCTTCGAACATCTGC |  |
|  | WsB_I100F_F | GATGTTCGAAAAACCTTTCTTAGTCACATC |  |
|  | WsB_I100F_R | GATGTGACTAAGAAAGGTTTTTCGAACATC |  |
|  | NdA_R98K_F100I_F | GCAGATGTCCGAAAAACCATTCTTAGTCACATC |  |
|  | NdA_R98K_F100I_R | GATGTGACTAAGAATGGTTTTTCGGACATCTGC |  |
|  | WsB_K228S_F | GATATCATTCTCACtcATGGAGGAATGAAGC |  |
|  | WsB_K228S_R | GCTTCATTCCTCCATgaGTGAGAATGATATC |  |
|  | WsB_R230C_F | CATTCTCACAAATGGTGTAATGAAGCAGAC |  |
|  | WsB_R230C_R | GTCTGCTTCATTACACCATTTGTGAGAATG |  |
|  | NdA_K234A_C236A_F | GATATCATTCTCACGCATGGGCCGATGAAGCGGAGATG |  |
|  | NdA_K234A_C236A_R | CATCTCCGCTTCATCGGCCCATGCGTGAGAATGATATC |  |
|  | NdA_G229A_Y230A_F | GGATGTGGCAACAATCGCCGCAGCTCATTCTCACAAATGGTGCG |  |
|  | NdA_G229A_Y230A_R | CGCACCATTTGTGAGAATGAGCTGCGGCGATTGTTGCCACATCC |  |
|  | NdA_K299L_F | TTGGGATTTGGGGACCGCCTGGGATTGGTCTGACGACCATCGCTG |  |
|  | NdA_K299L_R | CAGCGATGGTCGTCAGACCAATCCCAGGCGGTCCCCAAATCCCAA |  |
|  | NdA_L341A_F | ACGCACAATTGAAAGCACAAGAACAAATGTTG |  |
|  | NdA_L341A_R | CAACATTTGTTCTTGTGCTTTCAATTGTGCGT |  |
|  | NdA_M345A_F | GAAACTACAAGAACAAGCGTTGTCTCAGATTTTCAATC |  |
|  | NdA_M345A_R | GATTGAAAATCTGAGACAACGCTTGTTCTTGTAGTTTC |  |
|  | NdA_L346A_F | GAAACTACAAGAACAAATGGCGTCTCAGATTTTCAATC |  |
|  | NdA_L346A_R | GATTGAAAATCTGAGACGCCATTTGTTCTTGTAGTTTC |  |
|  | NdA_F350A_F | CAAATGTTGTCTCAGATTGCCAATCAGAAAGATACC |  |
|  | NdA_F350A_R | GGTATCTTTCTGATTGGCAATCTGAGACAACATTTG |  |
|  | NdA_I357A_F | CAGAAAGATACCATGGCTTCTCATTTAGGAGTG |  |
|  | NdA_I357A_R | CACTCCTAAATGAGAAGCCATGGTATCTTTCTG |  |
|  | NdA_S358A_F | CAGAAAGATACCATGATTGCTCATTTAGGAGTG |  |
|  | NdA_S358A_R | CACTCCTAAATGAGCAATCATGGTATCTTTCTG |  |
|  | NdA_E365A_F | GAGTGGCACCAGCACGTTTGAAAGAC |  |
|  | NdA_E365A_R | GTCTTTCAAACGTGCTGGTGCCACTC |  |
|  | NdA_E365I_F | AGGAGTGGCACCAATACGTTTGAAAGACAAG |  |
|  | NdA_E365I_R | CTTGTCTTTCAAACGTATTGGTGCCACTCCT |  |
|  | NdA_E365K_F | GAGTGGCACCAAAACGTTTGAAAGAC |  |
|  | NdA_E365K_R | GTCTTTCAAACGTTTTGGTGCCACTC |  |
|  | NdA_E365Q_F | GAGTGGCACCACAACGTTTGAAAGAC |  |
|  | NdA_E365Q_R | GTCTTTCAAACGTTGTGGTGCCACTC |  |
|  | NdA_V362A_F | CTCATTTAGGAGCGGCACCAGAACG |  |
|  | NdA_V362A_R | CGTTCTGGTGCCGCTCCTAAATGAG |  |

For primers used for cloning, numbers indicate the amino acid position included by the 5’ end of the primer. For site-directed mutagenesis primers, numbers indicate the amino acid position targeted for substitution.
